# D-serine supplementation is associated with mucosal-prioritized rumen development and propionate-enriched fermentation with selective microbial shifts in pre-weaning Hu lambs

**DOI:** 10.3389/fmicb.2026.1899537

**Published:** 2026-07-30

**Authors:** Shuoyi Zhang, Zhen Tang, Yanliao Ding, Peiyao Xu, Mireguli Yimamu, Kaixu Chen

**Affiliations:** Xinjiang Key Laboratory of Herbivore Nutrition for Meat & Milk, College of Animal Science, Xinjiang Agricultural University, Urumqi, China

**Keywords:** D-serine, metabolome, microbiome, pre-weaning lamb, ruminal development, ruminal fermentation

## Abstract

This study evaluated whether dietary D-serine (D-Ser) could modulate rumen development, fermentation, microbiota, and metabolomic profiles in pre-weaning Hu lambs. Twenty healthy male lambs were assigned to a control group or a D-Ser group (*n* = 10/group); D-Ser was supplied at 2 g·kg^−1^ BW·d^−1^ from 7 to 48 days of age. Growth and starter intake were recorded, and rumen morphology, volatile fatty acids, 16S rRNA profiles, and untargeted LC–MS metabolomes were analyzed in slaughtered lambs (*n* = 6/group). D-Ser increased average daily starter intake during the 30-d starter-intake recording period by 25.96% (*p* = 0.036) and tended to advance first voluntary starter intake, whereas final body weight and average daily gain were numerically but not significantly higher. Rumen weight, rumen weight-to-body weight ratio, and rumen volume were significantly increased. Papillae length, width, density, and epithelial thickness were also enhanced, while muscle layer thickness was unchanged, indicating mucosa-prioritized morphological development. D-Ser increased total volatile fatty acids, acetate, propionate, and butyrate concentrations; propionate molar proportion rose from 21.41 to 25.11%, and the acetate-to-propionate ratio decreased from 2.90 to 2.44. Microbial diversity was not significantly altered, but *Bacteroidota* abundance increased, with enrichment of *Shuttleworthia*, *Erysipelotrichaceae*_UCG-006/UCG-009, *Corynebacterium*, and *Sutterella*. Metabolomics identified 248 differential metabolites enriched in amino acid, carbohydrate, and secondary bile acid pathways. The upregulation of L-N-carboxymethylserine and isodeoxycholic acid was consistent with possible microbial processing of D-Ser, but direct transformation was not experimentally verified. Overall, D-Ser improved starter intake and was associated with propionate-enriched fermentation and mucosal morphological development, whereas its growth-promoting effect and causal microbial mechanisms require further validation.

## 1 Introduction

The preweaning period is a critical stage in which the rumen develops from a relatively immature foregut compartment into a functional fermentation organ. The coordinated maturation of ruminal papillae, epithelial barrier function, microbial colonization, and fermentation capacity determines starter feed utilization, weaning adaptation, and subsequent growth potential in young ruminants (Baldwin et al., 2004; Furman et al., 2020; Khan et al., 2016; Steele et al., 2016; Yáñez-Ruiz et al., 2015). Early-life nutritional interventions can redirect microbial colonization and fermentation end-product profiles. Increasing evidence also indicates that microbial metabolites mediate rumen epithelial development through microbiota-host interactions (Bian et al., 2024; Lin et al., 2019; Sander et al., 1959; Sun et al., 2024).

Rumen development is regulated by both physical and chemical stimuli. Solid feed provides mechanical stimulation that supports rumen volume expansion and muscular development, whereas volatile fatty acids (VFAs), particularly butyrate and propionate, stimulate papillae growth, epithelial cell proliferation, and the development of short-chain fatty acid transport capacity (Górka et al., 2011; Malhi et al., 2013; Niwińska et al., 2017; Penner et al., 2011). Practical strategies, including early starter feeding, sodium butyrate supplementation, and probiotic use, have therefore been applied to promote rumen maturation (Friedman, 1999; Wu, 2009, 2013). However, many current approaches target a single fermentation product or a restricted microbial response, and their ability to coordinate fermentation carbon flow, microbial metabolism, and mucosal development remains limited. Nutritional factors that improve fermentation efficiency while preserving microbial stability are still needed for precision feeding of young ruminants.

Amino acids are not only substrates for protein synthesis. They also participate in energy metabolism, redox balance, immune regulation, and cell signaling, which underpins the concept of functional amino acids in animal nutrition (Genchi, 2017; Lam et al., 2009). Compared with L-amino acids, D-amino acids have long been regarded as poorly utilized enantiomers, and their roles in ruminant nutrition remain underexplored (Aliashkevich et al., 2018; Cava et al., 2011; Mothet et al., 2000). Recent studies show that D-amino acids are not metabolically inert. In bacteria, they contribute to cell wall remodeling, biofilm formation, and community-level interactions, while D-serine has well-defined signaling functions in mammals (Diao et al., 2019; Malmuthuge et al., 2019; Sun et al., 2021; Zhang et al., 2022). Because the rumen is a high-density anaerobic microbial ecosystem enriched in amino acid transformation and microbial protein synthesis, it may provide an ecological niche in which exogenous D-serine interacts with ruminal microorganisms and is associated with downstream metabolic changes (Khan et al., 2011; Sullivan and Feinn, 2012).

Accordingly, we hypothesized that D-serine supplementation during the preweaning period could modulate ruminal microbial composition and metabolic activity, shift VFA production patterns, and promote mucosal development and the transition to starter feed. To test this hypothesis, Hu ram lambs were supplemented with an exploratory D-serine dose of 2 g·kg^−1^ BW·d^−1^ from 7 to 48 days of age. Growth performance, starter feed intake, rumen morphology, VFA concentrations, 16S rRNA microbial profiles, and untargeted metabolomic profiles of rumen fluid were integrated to evaluate the effects of D-serine on rumen development, fermentation, and microbiota-metabolite associations.

## 2 Materials and methods

### 2.1 Ethics

All animal husbandry, oral gavage, blood-sampling, and slaughter procedures performed in this trial were conducted in strict accordance with the welfare and use guidelines for laboratory animals issued by the Institutional Animal Care and Use Committee (IACUC) of Xinjiang Agricultural University, and were reviewed and approved by the same committee (approval code: 2023038). The experimental design, operational procedures, and reporting of results complied with the ARRIVE 2.0 guidelines.

### 2.2 Experimental materials

D-Serine (D-Ser; HPLC grade, chromatographic purity ≥ 99%) was purchased from Wuhan Jixinyibang Biotechnology Co., Ltd. (Wuhan, Hubei, China). Male Hu lambs (*Ovis aries*) were provided by Xinjiang Huaxing Sheep Farm (Xinjiang, China). The milk replacer was supplied by Beijing Precision Animal Nutrition Research Center Co., Ltd. (Beijing, China; product standard no. Q/HDJZA0004-2023), and the lamb starter feed was supplied by Xinjiang Sanwang Feed Co., Ltd. (Xinjiang, China; product standard no. Q/XSW003-2023).

### 2.3 Experimental animals and design

A single-factor completely randomized design was adopted, and the trial was conducted at Xinjiang Huaxing Sheep Farm from June to August 2024. Twenty healthy male Hu lambs born to multiparous ewes of identical parity (2nd–3rd parity), with synchronized lambing dates (± 3 d), comparable body-condition scores, and sound general health, were selected as the experimental animals on the basis of comparable birth weights. At 7 d of age, the initial body weight (IBW) of each lamb was recorded; the lambs were then ranked by IBW in ascending order and stratified-randomly assigned to either the control group (Group C) or the D-serine group (Group D) by means of a random-number table, with 10 lambs per group. To minimize operational bias, a single-blinded protocol was implemented in which sample collection and laboratory analyses were carried out independently by personnel who had not participated in group allocation and who were blinded to treatment assignment.

After parturition, all lambs were allowed to suckle naturally from their dams for the first 3 d to ensure adequate colostrum intake. To reduce pre-allocation variation, only lambs born to ewes with comparable parity, body-condition score, and general health were enrolled; however, individual ewe milk yield during these 3 d was not quantified, and residual variation in early milk intake cannot be completely excluded. From day 4 onward, the lambs were separated from their dams, transferred to artificial-rearing pens, and centrally fed with a standardized milk-replacer gavage regimen. Lambs in the treatment group (Group D) received D-serine at 2 g·kg^−1^ BW·d^−1^. This dose level corresponds to that used in our group’s previous study in suckling Hu lambs (Zhang et al., 2022), in which the same per-kilogram dose was well tolerated and significantly improved average daily gain and plasma GH and IGF-I; in the absence of established dose–response guidelines for ruminal D-serine supplementation, it was adopted here to maintain comparability with that work while extending the endpoints to rumen development and the microbiota-metabolome axis. It should therefore be interpreted as an exploratory, pharmacological-level intervention rather than an optimized recommended feeding dose. The D-serine was dissolved in the reconstituted milk replacer and administered in three equal aliquots per day. The lambs were weighed under fasting conditions at the beginning of each week, and the weekly allowances of D-serine and milk replacer were adjusted dynamically on this basis. Lambs in the control group (Group C) received equivalent volumes of D-serine-free milk replacer at matched time points. The total experimental duration was 45 d, comprising a 3-d pre-feeding period (days 4–6) for bottle-feeding adaptation and a 42-d formal feeding period (days 7–48), during which D-serine was actively administered.

At the conclusion of the formal feeding period, six lambs whose fasted body weights most closely approximated the group mean were selected from each group (*n* = 6) and subjected to a 12-h pre-slaughter feed-withdrawal period (with ad libitum access to water). In accordance with animal-welfare standards, these lambs were stunned by electrical immobilization and then exsanguinated via the carotid artery. The remaining four lambs in each group were not slaughtered and continued to be reared under the original management protocol. Accordingly, all *in vivo* indices (IBW, weekly BW, final body weight (FBW), total weight gain (TWG), average daily gain (ADG), and the dry-matter (DM) intake of milk replacer and starter feed) were determined in all 20 lambs (*n* = 10 per group), whereas all post-slaughter sampling indices (16S rRNA gene sequencing and untargeted metabolomic analysis of the rumen microbiota) were obtained from the 12 selected lambs (*n* = 6 per group).

### 2.4 Animal management and diet composition

The daily allowance of milk replacer was calculated as 2% of the current fasted body weight on a DM basis and was reconstituted with warm water (50 °C) at a feed-to-water ratio of 1:5. Once the temperature had decreased to 38–40 °C, the milk replacer was administered by dedicated personnel via oral gavage in three equal meals per day; the supplied amount was recalculated weekly on the basis of fasted body-weight measurements. Throughout gavage, the lambs were closely monitored for swallowing and respiratory reactions in order to prevent aspiration. For the treatment group, D-serine was accurately weighed at 2 g·kg^−1^ BW·d^−1^, added to the reconstituted milk replacer, and completely dissolved by means of a vortex mixer. The lambs were fed three times daily at 08:00, 14:00, and 20:00 and had ad libitum access to fresh drinking water throughout the trial.

From 15 days of age onward, the lamb starter feed was introduced gradually and offered separately from the milk replacer. The milk-replacer allowance was not decreased after starter feed introduction, and all lambs continued to receive the scheduled milk replacer until day 48. Thus, milk replacer and starter feed were provided concurrently during the starter-feeding period. The actual daily gavage volume of milk replacer and the daily refusal of starter feed were recorded. The daily starter-feed intake (DM basis) was calculated as the difference between the amounts offered and refused, and was recorded continuously until the end of the trial.

Ambient temperature in the sheep house was maintained at 18–24 °C, and relative humidity at 55–65% (recorded thrice daily—morning, noon, and evening). Natural daylight supplemented by artificial lighting was used to maintain a 12-h photoperiod, and daily ventilation was not less than 4 h. The bedding was replaced every 3 d, and disinfection was performed at regular intervals in accordance with standard operating procedures. The lambs were vaccinated in line with the locally recommended immunization schedule. Throughout the trial, the feed intake, respiration, fecal characteristics, and coat condition of each lamb were monitored and recorded daily; no mortality or severe morbidity occurred. The ingredient compositions and nutrient levels of the milk replacer and the lamb starter feed are presented in Tables 1, 2, respectively.

**Table 1:** Ingredient composition and nutrient levels of the milk replacer (DM basis).

Items	Content (%)
Milk replacer ingredients	
Whole milk powder	65.00
High-protein whey powder	18.00
Whey protein concentrate (WPC)	2.00
Soy protein concentrate	10.00
L-Lysine	2.20
DL-Methionine	0.80
L-Threonine	1.00
NaCl	0.50
Trace mineral premix^1^	0.25
Vitamin premix^2^	0.25
Total	100.00
Nutrient level^3^	
Metabolizable energy, ME (MJ·kg^−1^)	18.73
Dry matter, DM (%)	94.11
Crude protein, CP (%)	23.56
Ether extract, EE (%)	12.35
Crude fiber, CF (%)	2.18
Ash (%)	8.33
Calcium, Ca (%)	1.16
Total phosphorus, P (%)	0.84

1 The trace mineral premix supplied (per kg of milk replacer): Cu, 8 mg; Fe, 60 mg; Zn, 80 mg; Mn, 40 mg; I, 1 mg; Se, 0.5 mg; Co, 0.8 mg.

2 The vitamin premix supplied (per kg of milk replacer): vitamin A, 25,000 IU; vitamin D₃, 5,200 IU; vitamin E, 40 IU.

3 The ME value was calculated, whereas the remaining nutrient indices were measured on a DM basis from three replicate analyses (*n* = 3).

**Table 2:** Ingredient composition and nutrient levels of the lamb starter feed (DM basis).

Items	Content (%)
Starter feed ingredients	
Corn	62.80
Soybean meal	26.20
Wheat bran	7.00
CaHPO₄	1.00
Limestone	1.50
NaCl	0.50
Premix ^1^	1.00
Total	100.00
Nutrient level^2^	
Metabolizable energy, ME (MJ·kg^−1^)	12.83
Dry matter, DM (%)	83.16
Crude protein, CP (%)	18.67
Ether extract, EE (%)	3.42
Crude fiber, CF (%)	8.69
Ash (%)	8.78
Calcium, Ca (%)	1.46
Total phosphorus, P (%)	0.72

1 The premix supplied (per kg of starter feed): Cu, 10 mg; Fe, 80 mg; Zn, 100 mg; Mn, 50 mg; I, 0.8 mg; Se, 0.3 mg; Co, 0.5 mg; vitamin A, 15,000 IU; vitamin D₃, 3,000 IU; vitamin E, 50 IU.

2 The ME value was calculated, whereas the remaining nutrient indices were measured on a DM basis from three replicate analyses (*n* = 3).

### 2.5 Sample collection

On the final day of the formal feeding period (day 48), six lambs whose body weights most closely approximated the group mean were selected from each group (*n* = 6); after a 12-h fast, they were stunned by electrical immobilization and exsanguinated via the carotid artery by dedicated personnel in accordance with animal-welfare standards. Immediately after slaughter, the abdominal cavity was opened and the entire rumen was excised. An incision was made along the dorsoventral border of the ventral sac, and the ruminal contents were filtered through four layers of sterile gauze. Approximately 50 mL of rumen fluid per lamb was collected, rapidly aliquoted into 2-mL sterile cryogenic tubes, snap-frozen in liquid nitrogen, and transferred to a − 80 °C freezer for subsequent 16S rRNA gene high-throughput sequencing and liquid chromatography–mass spectrometry (LC–MS)-based untargeted metabolomic analysis. All pre- and post-slaughter procedures were performed by the same trained team in order to avoid selection bias.

### 2.6 Measurement parameters and methods

#### 2.6.1 Growth performance and feed intake

Before the start of the formal feeding period and every Monday morning thereafter prior to morning feeding, all lambs (*n* = 10 per group) were weighed under fasting conditions using an electronic floor scale (precision, 0.01 kg; model YH-T7, Shanghai Yingzhan Industrial Co., Ltd., Shanghai, China). The IBW (at day 7), FBW (at day 48), TWG, and ADG were recorded. The daily offered and refused amounts of milk replacer were accurately weighed and converted to DM intake. From 15 days of age onward, the previous day’s refusal of starter feed was weighed and recorded for each lamb at 07:30 daily; the daily starter-feed intake (as-fed basis) was calculated as the difference between the amounts offered and refused, and was subsequently converted to DM intake (DMI, g·d^−1^) using the DM content of the starter feed (83.16%). The age at first voluntary solid-feed intake and the cumulative starter-feed DM intake during the 30-d starter-intake recording period were also recorded for each lamb.

#### 2.6.2 Rumen development indices

After slaughter, the rumen was completely dissected, its contents were drained, and the ruminal wall was rinsed with 0.9% physiological saline. After being blotted dry with filter paper, the empty rumen tissue was weighed using an electronic balance with a precision of 0.1 g (model ME2002, Mettler-Toledo, Greifensee, Switzerland), and the ratio of rumen weight to live body weight (g·kg^−1^) was calculated. The rumen volume was determined by the water-filling method: after ligation of all ruminal orifices, warm water (38 °C) was injected until the ruminal wall was fully extended without distension, and the volume injected (mL) was recorded as the rumen volume.

A tissue block of approximately 2 × 2 cm^2^ was excised from the central region of the ventral sac, fixed in 4% paraformaldehyde for 24 h, dehydrated through a graded ethanol series, cleared in xylene, and embedded in paraffin. Sections (5-μm thickness) were then prepared and stained with hematoxylin and eosin (H&E). The sections were examined under a light microscope (model CX23, Olympus Corp., Tokyo, Japan), and Image-Pro Plus 6.0 software (Media Cybernetics, Inc., Rockville, MD, USA) was used to measure the papilla length, papilla width (at the base), total epithelial thickness, and muscle-layer thickness. For each lamb, at least 10 intact papillae were measured, and the mean value was taken as the individual datum; the papilla density was determined by counting the number of papillae within a 1 × 1 cm^2^ demarcated area.

#### 2.6.3 Determination of ruminal volatile fatty acids

The supernatant of the rumen fluid was filtered through a 0.22-μm membrane and analyzed for volatile fatty acids (VFAs) by gas chromatography coupled with flame ionization detection (GC–FID; model 7890B, Agilent Technologies, Santa Clara, CA, USA). Chromatographic separation was performed on an HP-FFAP capillary column (30 m × 0.25 mm × 0.25 μm; Agilent Technologies, Santa Clara, CA, USA); the injector and detector temperatures were maintained at 250 °C and 280 °C, respectively. The oven temperature program was as follows: an initial temperature of 100 °C held for 1 min; ramped at 10 °C·min^−1^ to 180 °C and held for 3 min; then ramped at 20 °C·min^−1^ to 220 °C and held for 2 min. High-purity nitrogen (≥ 99.999%) served as the carrier gas at a flow rate of 1.0 mL·min^−1^, with a split ratio of 10:1 and an injection volume of 1 μL. With 2-ethylbutyric acid as the internal standard, acetate, propionate, butyrate, isobutyrate, valerate, isovalerate, and 2-methylbutyrate were quantified using the external-standard method coupled with internal-standard correction. VFA standards were purchased from Sigma-Aldrich (St. Louis, MO, USA); the calibration range was 0.1–20 mmol·L^−1^, with *R*^2^ ≥ 0.999 for each individual VFA. The results are expressed both as absolute concentrations (mmol·L^−1^) and as molar proportions of the total VFA (TVFA; mol%); the acetate-to-propionate ratio (A/P) was also calculated.

#### 2.6.4 High-throughput sequencing of the ruminal 16S rRNA gene

Rumen-fluid samples (*n* = 6 per group; 12 samples in total) were submitted to Novogene Bioinformatics Technology Co., Ltd. (Beijing, China) for 16S rRNA gene sequencing. Genomic DNA was extracted by the cetyltrimethylammonium bromide (CTAB) method, and the DNA concentration and purity were assessed using a NanoDrop 2000 microvolume spectrophotometer (Thermo Fisher Scientific, Waltham, MA, USA) and verified by 1% agarose gel electrophoresis. The V3–V4 hypervariable region of the 16S rRNA gene was PCR-amplified using the bacterial-specific primer pair 341F (5′-CCTAYGGGRBGCASCAG-3′) and 806R (5′-GGACTACNVGGGTWTCTAAT-3′).

The 30-μL PCR reaction system comprised 15 μL of Phusion® High-Fidelity PCR Master Mix (New England Biolabs, Ipswich, MA, USA); 1 μL each of the forward and reverse primers (0.2 μmol·L^−1^); 10 ng of template DNA; and sterile deionized water to volume. The thermal cycling program comprised initial denaturation at 98 °C for 1 min; 30 cycles of denaturation at 98 °C for 10 s, annealing at 50 °C for 30 s, and extension at 72 °C for 30 s; and a final extension at 72 °C for 5 min. PCR products were checked by 2% agarose gel electrophoresis, purified using AMPure XP magnetic beads (Beckman Coulter, Inc., Brea, CA, USA), and pooled in equimolar concentrations. The sequencing libraries were constructed using the TruSeq® DNA PCR-Free Library Preparation Kit (Illumina, Inc., San Diego, CA, USA); library quality was verified with a Qubit 4.0 fluorometer (Thermo Fisher Scientific, Waltham, MA, USA) and quantitative PCR (model 7,500, Applied Biosystems, Foster City, CA, USA). Qualified libraries were then sequenced on the Illumina NovaSeq 6,000 platform using a 2 × 250-bp paired-end strategy.

Raw sequencing reads were initially processed with Cutadapt v1.9.1 to trim primer and adapter sequences. Paired-end reads were merged into raw tags using FLASH v1.2.11; thereafter, fastp v0.23.1 was applied for stringent quality control (average quality score Q ≥ 20; length ≥ 200 bp) to obtain clean tags. Chimeras were subsequently removed by comparison against the SILVA v138.1 database using the UCHIME algorithm (v7.0) to yield effective tags. Operational taxonomic units (OTUs) were clustered at a 97% similarity threshold using UPARSE v7.0.1001; in parallel, amplicon sequence variants (ASVs) were generated via the DADA2 pipeline implemented in QIIME2 v2023.9, and cross-validation of *α*- and *β*-diversity results indicated that the trends derived from the two pipelines were concordant. Representative sequences were taxonomically annotated against the SILVA v138.1 database.

α-Diversity indices (Chao1, observed_species, Shannon, Simpson, and Good’s coverage) were calculated using the feature-table plugin in QIIME2. β-Diversity was visualized by principal coordinate analysis (PCoA) based on Bray–Curtis distance matrices; the significance of intergroup differences was assessed by permutational multivariate analysis of variance (PERMANOVA; 999 permutations), and the homogeneity of multivariate dispersion was tested by PERMDISP. For LEfSe analysis, the significance level was set at α = 0.05 for both the Kruskal–Wallis non-parametric test and the Wilcoxon rank-sum test, and the linear discriminant analysis (LDA) score threshold was set at > 2.0; both raw *p*-values and LDA scores are reported to control for false positives. Microbial metabolic-pathway functions were predicted against the MetaCyc database using PICRUSt2 v2.5.2 after correction for 16S gene copy number; differential clusters of orthologous proteins were further annotated using the COG database. It should be emphasized that the 16S rRNA-based functional prediction provided by PICRUSt2 constitutes inferential functional annotation, whose precision is inferior to that of metagenomic sequencing; accordingly, all interpretations in the present study are restricted to the level of “predicted metabolic pathways” and should not be regarded as experimental validation of the actual metabolic function.

#### 2.6.5 Untargeted metabolomic analysis of the rumen fluid

Rumen-fluid samples (*n* = 6 per group; 12 samples in total) were submitted to Novogene Bioinformatics Technology Co., Ltd. (Beijing, China) for LC–MS-based untargeted metabolomic analysis.

(1) Sample preparation.

A 100-μL aliquot of rumen fluid was transferred into a 1.5-mL centrifuge tube; 400 μL of pre-chilled (−20 °C) 80% aqueous methanol containing the isotopic internal standard L-2-chlorophenylalanine (1 μg·mL^−1^) was then added. After vortexing for 30 s, the mixture was incubated on ice for 5 min and centrifuged at 15,000 × g and 4 °C for 20 min. A 200-μL aliquot of the supernatant was mixed with 200 μL of LC–MS-grade water—yielding a final methanol concentration of 53%—and centrifuged again at 15,000 × g and 4 °C for 20 min; the resulting supernatant was used for LC–MS analysis. A quality-control (QC) sample, prepared by pooling equal volumes of supernatant from all samples, was injected after every 10 samples to monitor instrument stability and data quality.

(2) LC–MS parameters.

Analyses were performed on a Vanquish UHPLC system coupled to a Q Exactive™ HF-X high-resolution mass spectrometer (Thermo Fisher Scientific, Bremen, Germany). Chromatographic separation was achieved on a Hypersil Gold C₁₈ column (100 mm × 2.1 mm, 1.9 μm; Thermo Fisher Scientific, Waltham, MA, USA) maintained at 40 °C, with a flow rate of 0.2 mL·min^−1^ and an injection volume of 2 μL. Under positive-ion mode, mobile phase A consisted of 10 mmol·L^−1^ aqueous ammonium acetate containing 0.1% formic acid and 95% acetonitrile, and mobile phase B consisted of 10 mmol·L^−1^ ammonium acetate containing 0.1% formic acid and 50% acetonitrile; under negative-ion mode, mobile phase A consisted of 10 mmol·L^−1^ ammonium acetate containing 95% acetonitrile, and mobile phase B consisted of 10 mmol·L^−1^ ammonium acetate containing 50% acetonitrile. The gradient-elution program was as follows: 0–12 min, B 2% → 100%; 12–14 min, B 100%; 14–17 min, B 100% → 2%. The mass-spectrometric parameters were as follows: scan range, m/z 100–1,500; spray voltage, +3.5 kV (positive-ion mode) and −3.0 kV (negative-ion mode); sheath-gas flow rate, 241 kPa (35 psi); auxiliary-gas flow rate, 10 L·min^−1^; ion-transfer-tube temperature, 320 °C; auxiliary-gas heater temperature, 350 °C; and S-lens RF level, 60. Data-dependent acquisition (DDA) mode was employed, with full-MS and MS/MS resolutions of 70,000 and 17,500, respectively.

(3) Data processing and metabolite identification.

Raw data files (.raw) were converted into mzXML format using ProteoWizard v3.0, and peak extraction, retention-time alignment, and peak alignment were carried out with XCMS v3.16 (mass deviation ≤ 10 ppm; retention-time deviation ≤ 0.15 min). Metabolites were identified by matching the accurate molecular masses (≤ 10 ppm), MS/MS fragmentation patterns, and retention times against the mzCloud, mzVault, HMDB v5.0, KEGG, and LipidMaps databases. The confidence level of metabolite identification was classified according to the Metabolomics Standards Initiative (MSI) as Level 1 (standard-verified), Level 2 (database-matched), or Level 3 (chemical-class-identified). It should be noted that several key differential metabolites in the present study (e.g., L-N-carboxymethylserine and isodeoxycholic acid) were not validated against authentic standards and therefore constitute putative identifications; this caveat has been taken into account when interpreting the results. After background-ion subtraction using the blank control, raw quantitative values were normalized within each batch according to the following equation (Equation 1):


$$\text{Relative Peak}\;\mathrm{Are}a_{\mathrm{ij}}=[x_{\mathrm{ij}}/\sum_{k=1}^{m}X_{\mathrm{ik}}]\times \sum_{k=1}^{m}X_{\mathrm{QC}1,k}$$ (1)

where xij denotes the raw peak area of metabolite j in sample i; m represents the total number of metabolites in the sample; and ΣxQC1,k denotes the sum of the peak areas of all metabolites in the first QC sample. After normalization, metabolites for which the coefficient of variation (CV) in the QC samples exceeded 30% were excluded in order to ensure data robustness.

(4) Statistical analysis of the metabolomic data.

After log₂ transformation and Pareto centring, principal component analysis (PCA) and orthogonal partial least squares-discriminant analysis (OPLS-DA) were performed using metaX v1.4.16 in R v4.3.1 (R Foundation for Statistical Computing, Vienna, Austria). The robustness of the OPLS-DA model was evaluated by 7-fold cross-validation and 200 permutation tests, with *R*^2^X, *R*^2^Y, *Q*^2^, and the *Q*^2^-intercept derived from permutation testing being reported (acceptance criteria: *R*^2^Y > 0.5; *Q*^2^ > 0.4; permutation *Q*^2^-intercept < 0.05). Differential metabolites were screened on the basis of three concurrent criteria—variable importance in projection (VIP) > 1; *p* < 0.05; and fold change (FC) > 1.5 or FC < 2/3 (i.e., FC < 0.667). Volcano plots integrating the VIP values, log₂(FC), and −log₁₀(*p*-value) of the individual metabolites were generated with the ggplot2 package in R. Functional annotation and pathway-enrichment analyses of the differential metabolites were performed against the KEGG database; the statistical significance of pathway enrichment was assessed by hypergeometric testing, with a pathway considered significantly enriched (*p* < 0.05) when the proportion of differential metabolites in the target pathway (x/n) was significantly higher than its theoretical proportion among all metabolites (y/N). All bioinformatic analyses were performed on the NovoMagic Cloud platform (Novogene).^1^

### 2.7 Correlation analysis

Given that 16S rRNA gene-derived relative-abundance data are compositional in nature, genus-level relative abundances were first subjected to centered log-ratio (CLR) transformation; thereafter, Spearman’s rank correlation coefficient was used to evaluate the associations between the ruminal microbiota (LEfSe-identified differential genera and core dominant genera) and the differential metabolites. Correlation heatmaps were generated on the Metware Cloud platform (Metware Biotechnology Co., Ltd., Wuhan, China),^2^ and the asterisks “*,” “**,” and “***” denote significance levels of *p* < 0.05, *p* < 0.01, and *p* < 0.001, respectively.

### 2.8 Data processing and statistical analysis

Experimental data were initially compiled in Microsoft Excel 2019 (Microsoft Corp., Redmond, WA, USA); statistical analyses were performed using SPSS v27.0 (IBM Corp., Armonk, NY, USA) and GraphPad Prism v10.0.0 (GraphPad Software Inc., San Diego, CA, USA), whereas high-dimensional omics data analyses and visualizations were carried out in R v4.3.1. *In vivo* measurements (IBW, FBW, weekly BW, TWG, ADG, and the DM intake of both milk replacer and starter feed) were obtained from all 20 lambs (*n* = 10 per group), whereas post-slaughter measurements (16S rRNA gene sequencing and untargeted metabolomic analyses of the rumen microbiota) were obtained from the 12 selected lambs (*n* = 6 per group); the sample size for each analysis is explicitly stated in the corresponding tables and figure legends.

For continuous variables, the Shapiro–Wilk test was first applied to assess normality, and Levene’s test was used to evaluate the homogeneity of variances. Variables satisfying both assumptions were compared between the groups using the independent-samples Student’s *t*-test, whereas those that failed to meet these assumptions were analyzed by the Mann–Whitney U test. The growth-performance indices (FBW, TWG, and ADG) were analyzed by analysis of covariance (ANCOVA) with IBW as the covariate; covariate-adjusted least squares means (LSM) ± the standard error of the mean (SEM) are reported. For the weekly repeated-measures body-weight data, a linear mixed model (LMM) was employed, in which treatment, time, and their interaction were specified as fixed effects, individual lamb was specified as a random effect, and the optimal covariance structure was selected from the unstructured (UN), first-order autoregressive (AR1), and compound-symmetric (CS) forms on the basis of the Akaike information criterion (AIC).

All data are presented as mean ± standard deviation (mean ± SD); for key indices such as growth performance, the effect size (Cohen’s d) and its 95% confidence interval are additionally reported. Given the limited sample size of the present study, *post hoc* power analyses were conducted on the growth indices in order to evaluate the statistical power of the present design to detect the observed effects. Statistical significance was set at *p* < 0.05, while 0.05 ≤ *p* < 0.10 was regarded as a statistical tendency; the term “trend” is used with caution in the Discussion in order to avoid over-interpretation. The statistical model is given by Equation 2:


$$Y_{\mathrm{ij}}=\mu +T_{i}+\beta X_{\mathrm{ij}}+\varepsilon_{\mathrm{ij}}$$ (2)

where Yij denotes the dependent variable (e.g., ADG or the relative abundance of a ruminal microbial taxon); μ is the overall mean; Ti is the fixed effect of the treatment group (i = C, D); Xij is the covariate (IBW); β is the regression coefficient of the covariate; and εij is the random residual, with εij ~ N(0, σ^2^).

## 3 Results

### 3.1 Effects of D-serine on growth performance and starter feed intake

As shown in Table 3, the initial body weight was identical between the C and D groups at the beginning of the trial (5.19 kg in both groups; *p* = 0.990), indicating comparable baseline conditions between treatments. After the 42-day feeding period, lambs supplemented with D-serine showed numerically higher final body weight, total weight gain, and average daily gain than the control lambs, with values of 13.92 kg, 8.74 kg, and 0.208 kg·d^−1^ in the D group compared with 11.52 kg, 6.33 kg, and 0.151 kg·d^−1^ in the C group, respectively. These corresponded to numerical increases of 20.83, 38.02, and 37.75%. However, after adjustment for initial body weight, none of these growth performance variables differed significantly between groups (*p* > 0.05). The Cohen’s d values for final body weight, total weight gain, and average daily gain were 0.47, 0.48, and 0.47, respectively, indicating numerical differences that do not support a statistically significant improvement in growth performance under the present sample size.

**Table 3:** Effects of D-serine on growth performance and starter feed intake in pre-weaning lambs.

Item	C group (*n* = 10)	D group (*n* = 10)	SEM	*p*-value	Cohen’s d
Initial body weight, IBW (kg)	5.19	5.19	0.36	0.990	0.01
Final body weight, FBW (kg)	11.52	13.92	1.62	0.328	0.47
Total weight gain, TWG (kg)	6.33	8.74	1.60	0.317	0.48
Average daily gain, ADG (kg/d)	0.151	0.208	0.038	0.317	0.47
Age at first voluntary starter intake (d)	19.8	18.2	0.64	0.092	0.80
Starter ADFI (g DM/d)	41.6	52.4	3.34	0.036	1.03
Cumulative starter intake (30-d recording period, g DM)	1,248	1,572	100.5	0.036	1.03
Feed conversion ratio, F: G	1.25	1.13	0.071	0.072	0.80

FBW, TWG, and ADG are reported as covariate-adjusted least-squares means (LSM) with pooled standard error of the mean (SEM), with IBW as the covariate in ANCOVA; the remaining variables are presented as group means with pooled SEM. Cohen’s d denotes the standardized effect size. Starter feed intake and F: G refer to cumulative values from the 30-d starter-intake recording period after starter feed introduction at 15 days of age. *p* < 0.05 was taken as significant and 0.05 ≤ P < 0.10 as a tendency.

The response in starter feed intake was more evident than that in body weight gain. Lambs in the D group began voluntary starter feed intake 1.6 days earlier than those in the C group (18.2 vs. 19.8 d), showing a tendency toward earlier solid feed acceptance (*p* = 0.092). During the 30-d starter-intake recording period, D-serine supplementation significantly increased average daily starter intake from 41.6 to 52.4 g DM·d^−1^ (*p* = 0.036), representing a 25.96% increase. Total starter feed intake was also higher in the D group than in the C group (1,572 vs. 1,248 g DM; *p* = 0.036). In addition, the feed-to-gain ratio was numerically reduced by 9.6% in the D group compared with the C group (1.13 vs. 1.25), with a tendency toward improvement (*p* = 0.072). These findings indicate that D-serine supplementation primarily enhanced starter feed acceptance and intake in pre-weaning lambs, which may support the nutritional transition from milk-based feeding to solid feed.

### 3.2 Effects of D-serine on rumen development

Rumen development indices are presented in Table 4. Body weight at slaughter did not differ between groups (13.85 vs. 12.36 kg, *p* = 0.346), confirming that the slaughtered animals were comparable in body size. At the tissue level, rumen fresh weight, the rumen-to-live-weight ratio, and rumen volume were all significantly greater in the D group (*p* = 0.011, 0.031, and 0.008), corresponding to increases of 22.04, 15.78, and 22.14% over the C group. Ventral-sac papillae length, papillae width, papillae density, and total epithelial thickness were likewise greater in the D group (*p* = 0.004, 0.019, 0.013, and 0.028); the largest relative gain was for papillae length (1,645 vs. 1,386 μm, +18.68%). In contrast, muscle layer thickness showed only a numerical increase (*p* = 0.154). Taken together, D-serine supplementation enhanced rumen mass, volumetric capacity, and papillae morphogenesis, with mucosal development clearly outpacing muscular development.

**Table 4:** Effects of dietary D-serine supplementation on rumen development of pre-weaning Hu lambs.

Item	C group (*n* = 6)	D group (*n* = 6)	*p*-value
Slaughter age (d)	48.0 ± 0.0	48.0 ± 0.0	—
Body weight at slaughter (kg)	12.36 ± 1.84	13.85 ± 1.96	0.346
Rumen fresh weight (g)	186.4 ± 28.7	227.2 ± 29.9	0.011
Rumen weight/body weight (g/kg)	15.08 ± 1.92	17.46 ± 1.67	0.031
Rumen volume (mL)	682 ± 98	833 ± 109	0.008
Papillae length, ventral sac (μm)	1,386 ± 214	1,645 ± 228	0.004
Papillae width, ventral sac (μm)	312 ± 41	367 ± 49	0.019
Papillae density, ventral sac (n/cm^2^)	58.2 ± 7.1	69.1 ± 8.8	0.013
Total epithelial thickness (μm)	98.4 ± 12.6	117.3 ± 16.4	0.028
Muscle layer thickness (μm)	486 ± 71	538 ± 78	0.154

Data are presented as mean ± SD. Rumen tissue parameters were measured after the rumen had been emptied of digesta. Papillae morphometric parameters were determined on H&E-stained ventral-sac sections, with ≥10 intact papillae measured per animal. Between-group comparisons were made by Student’s independent-samples *t*-test.

### 3.3 Effects of D-serine on rumen volatile fatty acids

Rumen volatile fatty acid (VFA) profiles are summarized in Table 5. Total VFA (TVFA) concentration was significantly higher in the D group than in the C group (77.42 vs. 64.28 mmol/L, *p* = 0.005). Absolute concentrations of acetate (C2), propionate (C3), and butyrate (C4) were also significantly elevated (*p* = 0.017, < 0.001, and 0.036), with relative increases of 17.84, 39.68, and 20.85%, respectively. On a molar basis, the propionate proportion rose from 21.41 to 25.11% (*p* = 0.003), the acetate proportion changed only marginally (61.98 to 61.06%, *p* = 0.181), and the acetate-to-propionate ratio (A/P) decreased from 2.90 to 2.44 (*p* = 0.005), indicating a moderate shift toward a propionate-enriched fermentation pattern. The butyrate molar proportion was unaffected (*p* = 0.857), as were the branched- and medium-chain fatty acids (isobutyrate, valerate, isovalerate, and 2-methylbutyrate; all *p* > 0.05).

**Table 5:** Effects of dietary D-serine supplementation on rumen volatile fatty acid concentrations and molar proportions in pre-weaning Hu lambs.

Item	C group (*n* = 6)	D group (*n* = 6)	*p*-value
TVFA (mmol/L)	64.28 ± 8.42	77.42 ± 8.86	0.005
Acetate (mmol/L)	39.84 ± 5.31	46.95 ± 6.08	0.017
Propionate (mmol/L)	13.76 ± 2.48	19.22 ± 3.62	<0.001
Butyrate (mmol/L)	7.42 ± 1.36	8.96 ± 1.64	0.036
Isobutyrate (mmol/L)	0.86 ± 0.18	0.71 ± 0.18	0.381
Valerate (mmol/L)	1.12 ± 0.24	1.45 ± 0.28	0.151
Isovalerate (mmol/L)	0.94 ± 0.21	1.05 ± 0.24	0.591
2-Methylbutyrate (mmol/L)	0.34 ± 0.08	0.48 ± 0.09	0.072
Acetate molar proportion (%)	61.98 ± 2.74	61.06 ± 2.49	0.181
Propionate molar proportion (%)	21.41 ± 2.13	25.11 ± 2.84	0.003
Butyrate molar proportion (%)	11.54 ± 1.38	11.67 ± 1.44	0.857
Acetate/propionate ratio	2.90 ± 0.36	2.44 ± 0.31	0.005

Data are presented as mean ± SD. *p*-values were obtained from Student’s independent-samples *t*-test after verifying normality and homogeneity of variance; molar proportions were arcsine-square-root transformed before analysis. *p* < 0.05 was taken as significant.

### 3.4 Effects of D-serine on rumen microbial composition and predicted function

#### 3.4.1 Microbial community structure and *α*/*β* diversity

**Figure 1:**
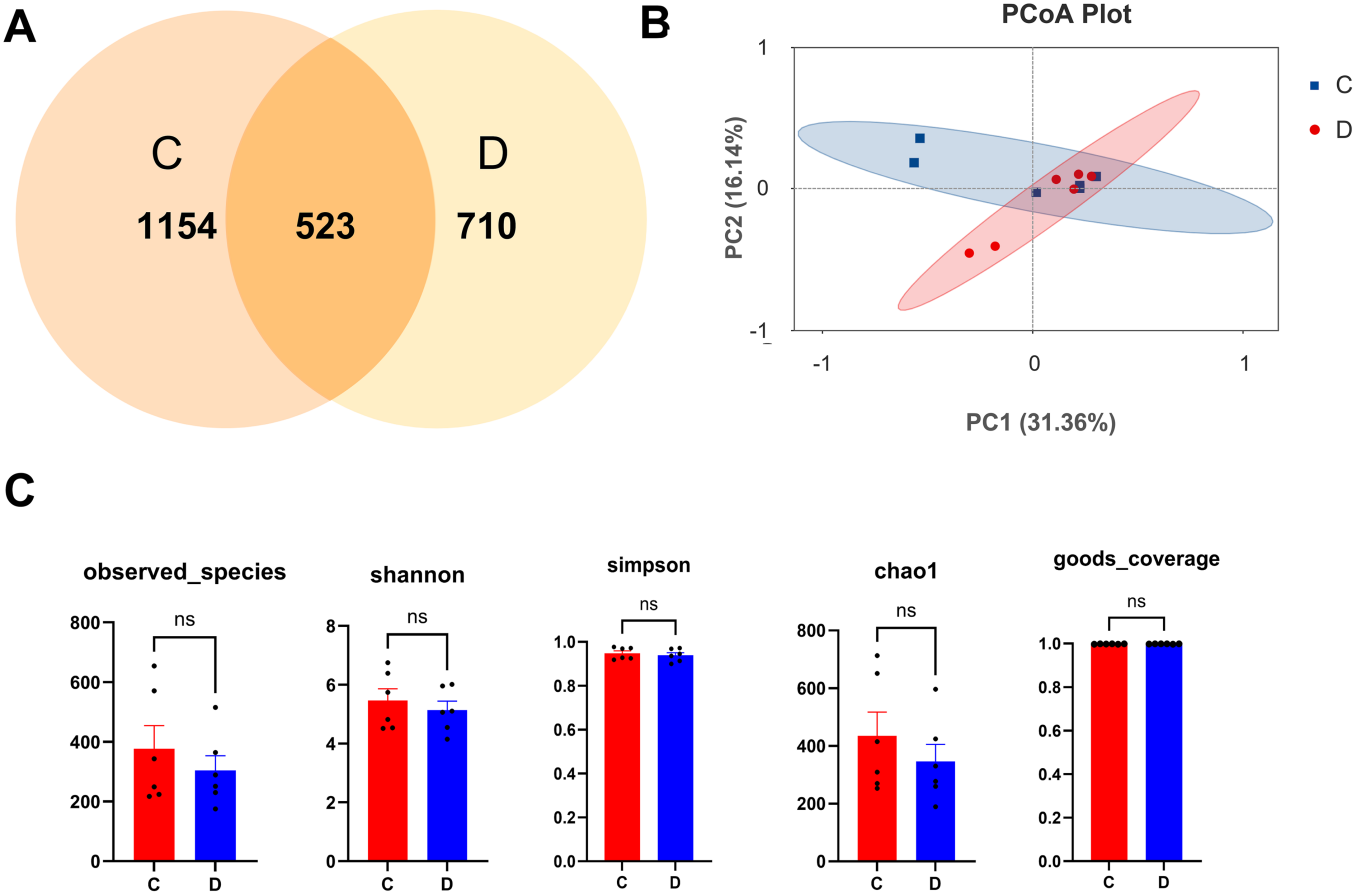
Effects of dietary D-serine supplementation on the rumen microbial community of pre-weaning Hu lambs. **(A)** Venn diagram of ASVs shared and unique to the C and D groups. **(B)** PCoA plot based on the Bray–Curtis distance matrix; ellipses denote 95% confidence regions. **(C)** α-diversity indices (observed species, Shannon, Simpson, Chao1, and Good’s coverage); data are mean ± SEM; ns, not significant (*p* > 0.05); the same notation applies hereafter.

High-throughput sequencing of the 16S rRNA gene V3–V4 region delineated the rumen microbial community of the two groups (Figure 1). The Venn diagram (Figure 1A) revealed 523 amplicon sequence variants (ASVs) shared between groups, together with 1,154 and 710 ASVs unique to the C and D groups, respectively. Principal coordinates analysis (PCoA) based on the Bray–Curtis distance matrix (Figure 1B) explained 31.36% (PC1) and 16.14% (PC2) of the total variance; the two clusters lay close together with partly overlapping 95% confidence ellipses, indicating broadly similar community structure. PERMANOVA (999 permutations) detected no significant between-group separation (*R*^2^ = 0.103, *p* = 0.146), and PERMDISP showed no difference in multivariate dispersion (*p* = 0.524). None of the *α*-diversity indices (observed species, Shannon, Simpson, Chao1, and Good’s coverage; Figure 1C) differed between groups (*p* > 0.05), indicating that D-serine at the dose used did not perturb overall microbial diversity.

#### 3.4.2 Microbial taxonomic composition

**Figure 2:**
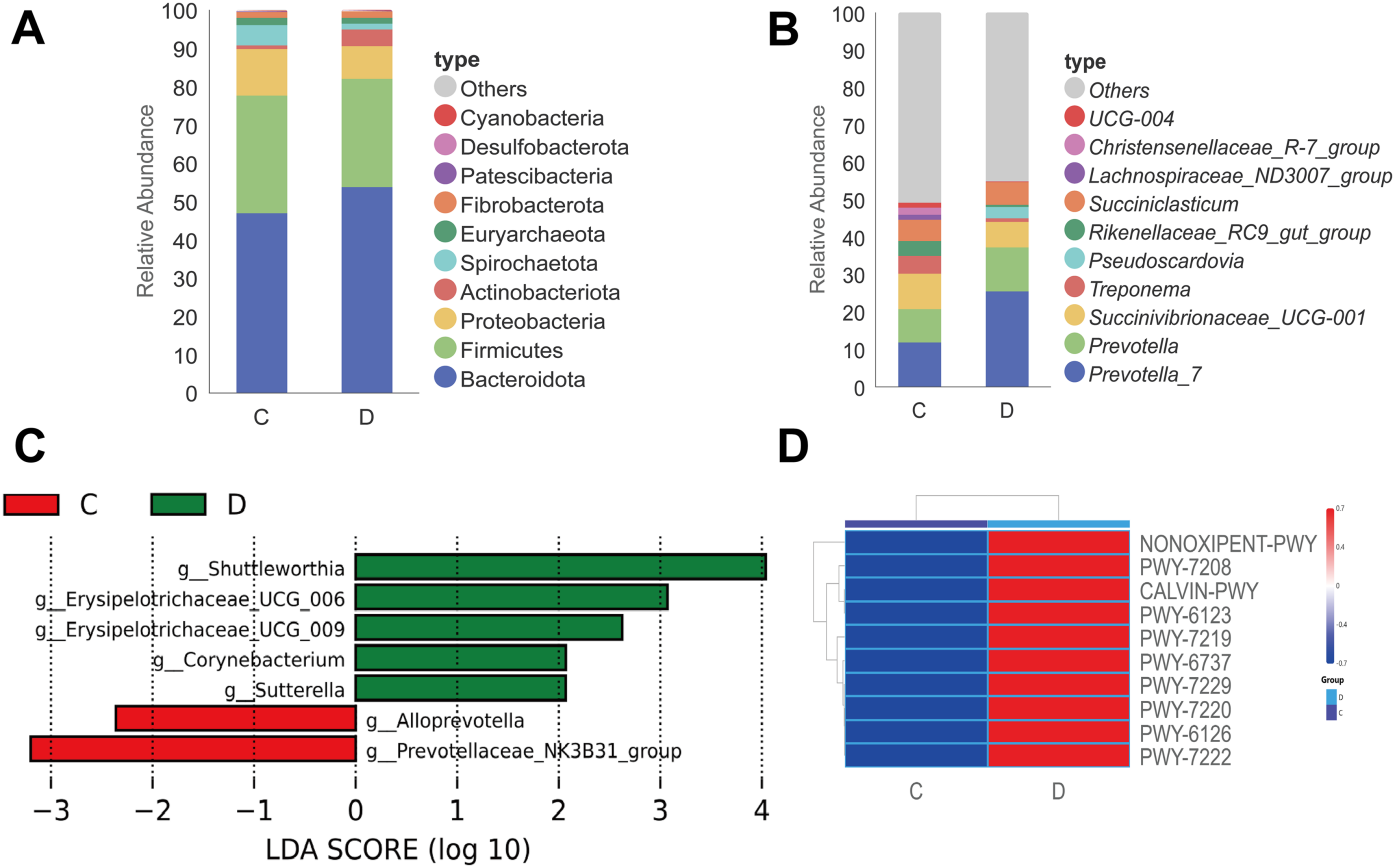
Effects of dietary D-serine supplementation on rumen microbial composition, differential biomarkers, and predicted function in pre-weaning Hu lambs. **(A)** Stacked bar plot of phylum-level relative abundance. **(B)** Stacked bar plot of the 10 most abundant genera. **(C)** LEfSe differential bar plot (LDA score threshold > 2.0; Kruskal–Wallis and Wilcoxon tests, *α* = 0.05); red and green bars mark biomarkers significantly enriched in the C and D groups, respectively. **(D)** Heatmap of MetaCyc Pathway Level 2 abundances predicted by PICRUSt2 (top 10 pathways elevated in the D group). The predicted functions represent inferential annotations from 16S rRNA sequences and should not be equated with experimentally verified metabolic activities.

At the phylum level (Figure 2A), the rumen community was dominated by *Bacteroidota*, *Firmicutes*, *Proteobacteria*, *Spirochaetota*, and *Actinobacteriota*; together, these five phyla accounted for 96.1% of sequences in the C group and 96.3% in the D group. The relative abundance of *Bacteroidota* was significantly higher in the D group than in the C group (52.92% vs. 46.27%, *p* = 0.031), whereas the other four dominant phyla did not differ between groups (*p* > 0.05). At the genus level (Figure 2B), none of the 10 most abundant genera (*Prevotella*, *Prevotella*_7, *Succinivibrionaceae*_UCG-001, *Treponema*, *Pseudoscardovia*, *Rikenellaceae*_RC9_gut_group, *Succiniclasticum*, *Lachnospiraceae*_ND3007_group, *Christensenellaceae*_R-7_group, and UCG-004) showed significant between-group differences (*p* > 0.05).

#### 3.4.3 LEfSe differential analysis

LEfSe analysis (Kruskal–Wallis and Wilcoxon non-parametric tests, α = 0.05; LDA score threshold > 2.0) identified seven differentially abundant biomarkers (Figure 2C). Five genus-level taxa were enriched in the D group: *Shuttleworthia* (LDA = 4.12, *p* = 0.018), *Erysipelotrichaceae*_UCG-006 (LDA = 3.06, *p* = 0.026), *Erysipelotrichaceae*_UCG-009 (LDA = 2.74, *p* = 0.035), *Corynebacterium* (LDA = 2.51, *p* = 0.041), and *Sutterella* (LDA = 2.38, *p* = 0.046). Two taxa were enriched in the C group: *Alloprevotella* (LDA = 2.83, *p* = 0.029) and *Prevotellaceae*_NK3B31_group (LDA = 3.47, *p* = 0.012). Thus, although D-serine left overall diversity unchanged, it was associated with limited taxonomic shifts at the phylum and genus levels rather than broad microbiota remodeling.

#### 3.4.4 Predicted microbial function (PICRUSt2/MetaCyc)

PICRUSt2 (v2.5.2) prediction against the MetaCyc database (Figure 2D) identified the 10 Pathway Level 2 categories with the highest predicted abundance in the D group relative to the C group. These categories spanned nucleoside/nucleotide biosynthesis, carbohydrate metabolism, and cofactor synthesis, and specifically included the non-oxidative pentose phosphate pathway (NONOXIPENT-PWY), pyrimidine deoxyribonucleotide phosphorylation (PWY-7208), the Calvin cycle (CALVIN-PWY), 5′-inosinate biosynthesis (PWY-6123), adenine-nucleotide biosynthesis pathways (PWY-7219, PWY-7229, and PWY-6126), a glycolysis/gluconeogenesis-related carbohydrate metabolism pathway (PWY-6737), hexitol fermentation to lactate, formate, ethanol, and acetate (PWY-7220), and glutaryl-CoA degradation (PWY-7222). These results are reported as predicted functional profiles derived from 16S rRNA data and should not be interpreted as directly measured pathway activities.

### 3.5 Effects of D-serine on the rumen metabolome

#### 3.5.1 Overview and classification of rumen metabolites

**Figure 3:**
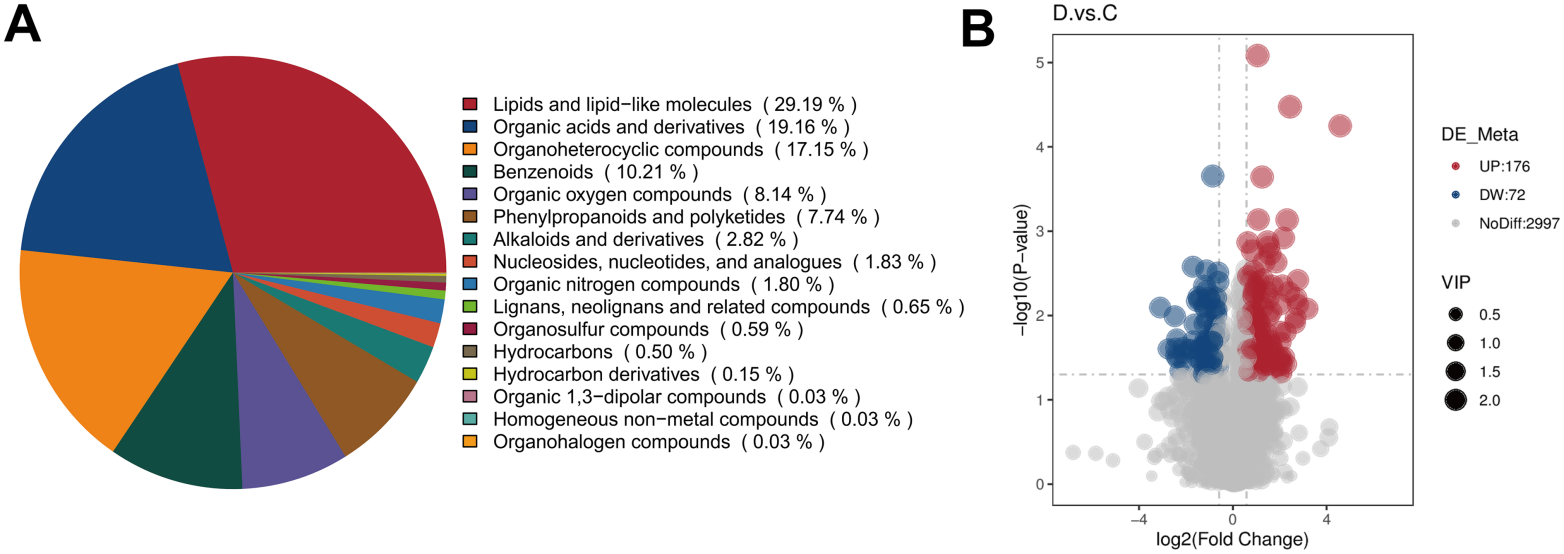
Effects of dietary D-serine supplementation on the rumen fluid metabolite profile of pre-weaning Hu lambs. **(A)** Pie chart of HMDB super-class classification (3,245 metabolites distributed across 16 chemical classes). **(B)** Volcano plot of differential metabolites between the D and C groups, with log2(fold change, D/C) on the x-axis and −log10(*p*-value) on the y-axis; red and blue dots mark metabolites significantly upregulated and downregulated in the D group, respectively (VIP > 1, P < 0.05, FC > 1.5); gray dots represent non-significant metabolites.

Untargeted metabolomic analysis on a UHPLC–Q Exactive HF-X platform yielded stable, reproducible signals: 93.7% of features exhibited a coefficient of variation (CV) below 30% in repeated injections of pooled quality control (QC) samples, and QC samples clustered tightly in the PCA score plot. A total of 3,245 metabolites were detected across the 12 rumen fluid samples (*n* = 6 per group). According to HMDB super-class classification (Figure 3A), the metabolites fell into 16 chemical classes, dominated by lipids and lipid-like molecules (29.19%), organic acids and derivatives (19.16%), organoheterocyclic compounds (17.15%), benzenoids (10.21%), organic oxygen compounds (8.14%), and phenylpropanoids and polyketides (7.74%); minor classes included alkaloids and derivatives (2.82%), nucleosides, nucleotides, and analogs (1.83%), organic nitrogen compounds (1.80%), lignans, neolignans, and related compounds (0.65%), organosulfur compounds (0.59%), hydrocarbons (0.50%), hydrocarbon derivatives (0.15%), and three remaining classes each accounting for 0.03% (organic 1,3-dipolar compounds, homogeneous non-metal compounds, and organohalogen compounds).

#### 3.5.2 OPLS-DA and screening of differential metabolites

Orthogonal partial least squares discriminant analysis (OPLS-DA) was used to compare the rumen metabolic profiles of the two groups. The model achieved *R*^2^X = 0.582, *R*^2^Y = 0.973, and *Q*^2^ = 0.812 in 7-fold cross-validation; a 200-permutation test yielded a *Q*^2^ intercept of −0.348 (< 0), confirming robustness without overfitting. The C and D groups were clearly separated in the OPLS-DA score plot. Applying a triple filter of VIP > 1, *p* < 0.05, and FC > 1.5 (i.e., FC > 1.5 or FC < 0.667), 248 differential metabolites were identified, of which 176 were upregulated and 72 downregulated in the D group; the remaining 2,997 metabolites were unchanged (Figure 3B, volcano plot).

#### 3.5.3 KEGG pathway enrichment

**Figure 4:**
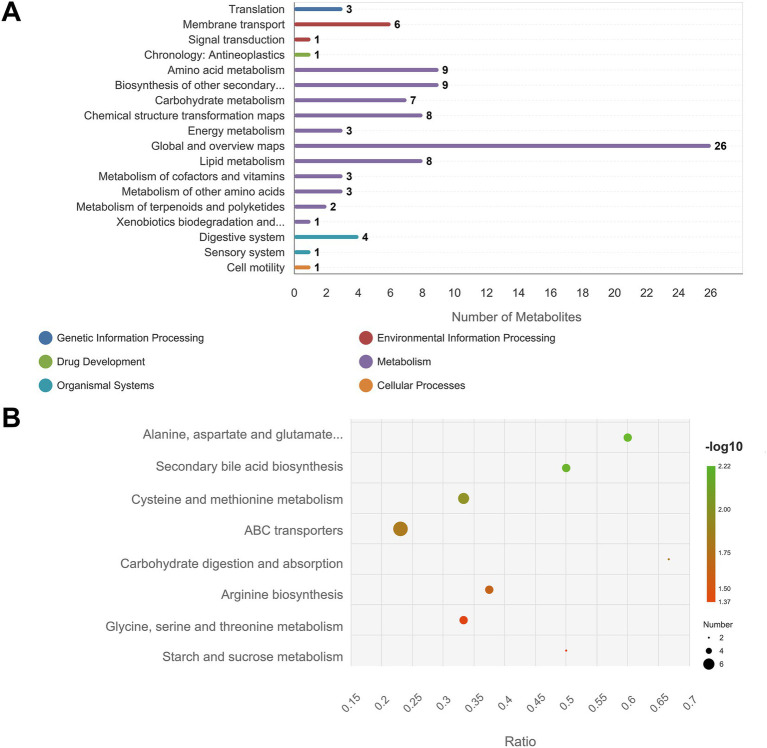
Effects of dietary D-serine supplementation on rumen metabolic pathways of pre-weaning Hu lambs. **(A)** Bar plot of KEGG pathway classification of differential metabolites, grouped by Pathway Level 1 functional category. **(B)** Bubble plot of KEGG pathway enrichment; the x-axis (Ratio) is the proportion of differential metabolites among all metabolites annotated to a given pathway (hits/background), the y-axis lists pathway names, bubble size is proportional to the number of differential metabolites in each pathway, and bubble color is scaled to −log10(*p*-value), where *p*-values are raw hypergeometric-test values without multiple-testing correction.

KEGG annotation of all 248 differential metabolites (Figure 4A) assigned them to six functional categories—metabolism (79.5%), environmental information processing, organismal systems, genetic information processing, drug development, and cellular processes. Within metabolism, amino acid metabolism, lipid metabolism, carbohydrate metabolism, and biosynthesis of other secondary metabolites were the most heavily represented subcategories.

Pathway enrichment analysis (hypergeometric test, *p* < 0.05) identified eight significantly enriched KEGG pathways (Figure 4B). Grouped by category, these were: amino acid metabolism—alanine, aspartate and glutamate metabolism (map00250), cysteine and methionine metabolism (map00270), arginine biosynthesis (map00220), and glycine, serine and threonine metabolism (map00260); energy and carbohydrate metabolism—carbohydrate digestion and absorption (map04973) and starch and sucrose metabolism (map00500); and two additional pathways, secondary bile acid biosynthesis (map00121) and ABC transporters (map02010).

#### 3.5.4 Key differential metabolites

The top 10 differential metabolites ranked by VIP value are listed in Table 6. Nine of the 10 were upregulated in the D group (log2FC > 0)—isodeoxycholic acid, L-N-carboxymethylserine, ferulic acid 4-O-sulfate, galanganal, (+)-desoxyscalarin, tigogenin, euscaphic acid, trans-resorcylide, and medicagenic acid—while only stereonsteroid A was downregulated (log2FC = −0.860). The two most strongly upregulated compounds, L-N-carboxymethylserine (log2FC = 4.576) and isodeoxycholic acid (log2FC = 2.437), provide a chemical fingerprint of microbial conversion of the supplemented D-serine.

**Table 6:** Top 10 differential rumen-fluid metabolites between the D and C groups, ranked by VIP value.

Metabolite	VIP	*p*-value	log2FC	Trend	CAS No.
Isodeoxycholic acid	2.446	0.000034	2.437	↑	566–17-6
L-N-carboxymethylserine	2.317	0.000056	4.576	↑	17,136–47-9
Stereonsteroid A	2.242	0.000222	−0.860	↓	NA
Ferulic acid 4-O-sulfate	2.308	0.000228	1.247	↑	151,481–53-7
Galanganal	2.121	0.000728	1.087	↑	NA
(+)-Desoxyscalarin	2.281	0.000734	2.312	↑	NA
Tigogenin	2.158	0.001205	2.161	↑	77–60-1
Euscaphic acid	2.187	0.001333	1.481	↑	53,155–25-2
trans-Resorcylide	2.136	0.001358	0.638	↑	NA
Medicagenic acid	2.125	0.001542	1.525	↑	599–07-5

VIP indicates the contribution of each metabolite to OPLS-DA group separation; *p*-values were obtained from Student’s independent-samples *t*-test; log2FC denotes the log2-transformed fold change (D/C); ↑ and ↓ indicate up- and downregulation in the D group, respectively; NA, CAS number not available in the queried databases. All metabolites listed correspond to MSI Level 2 putative identifications and have not been confirmed against authentic standards.

### 3.6 Correlation between differential genera and differential metabolites

**Figure 5:**
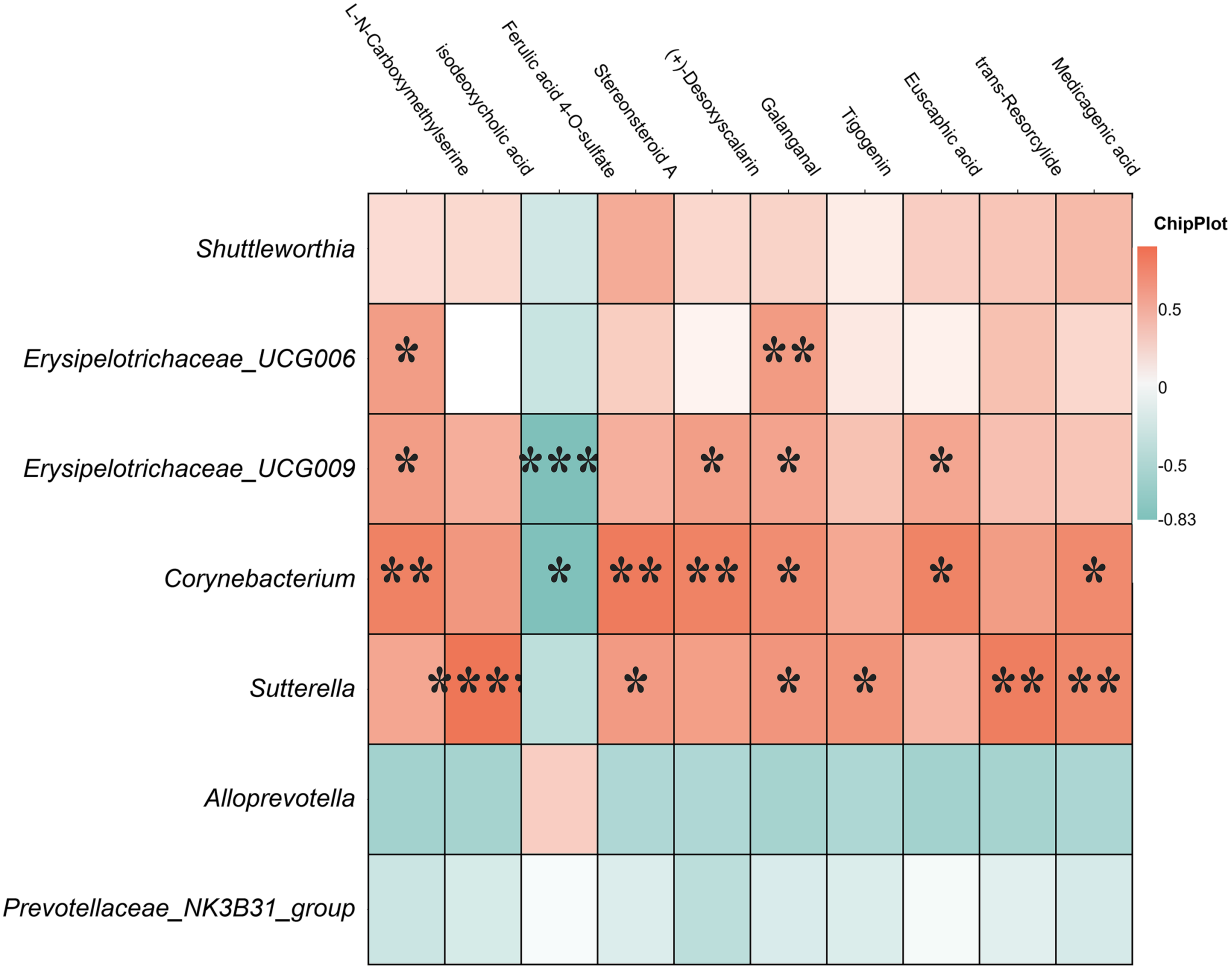
Spearman correlation heatmap of differential rumen genera with key differential metabolites. Genus-level relative abundances were CLR-transformed before correlation. The color scale (−0.83 to 0.5) encodes Spearman’s ρ; red and blue denote positive and negative correlations, respectively. Statistical significance is indicated by asterisks: *p < 0.05, **p < 0.01, ***p < 0.001.

Spearman rank correlation analysis was performed between the seven LEfSe-identified genus-level biomarkers and the top 10 differential metabolites listed in Table 6 after centered log-ratio (CLR) transformation of relative abundances; results are visualized as a heatmap in Figure 5. The five D-group-enriched genera (*Shuttleworthia*, *Erysipelotrichaceae*_UCG-006, *Erysipelotrichaceae*_UCG-009, *Corynebacterium*, and *Sutterella*) were positively correlated with all nine upregulated metabolites (isodeoxycholic acid, L-N-carboxymethylserine, ferulic acid 4-O-sulfate, galanganal, (+)-desoxyscalarin, tigogenin, euscaphic acid, trans-resorcylide, and medicagenic acid). *Corynebacterium* and *Sutterella* reached significance (*p* < 0.05) to high significance (*p* < 0.01) for multiple metabolites, and *Erysipelotrichaceae*_UCG-009 reached *p* < 0.001 against stereonsteroid A and several plant-derived metabolites. Among the C-group-enriched taxa, *Alloprevotella* tended to correlate positively with the downregulated stereonsteroid A (*ρ* = 0.43, *p* = 0.087) without reaching significance, while *Prevotellaceae*_NK3B31_group showed no significant correlation with any of the listed metabolites (*p* > 0.05). These coordinated patterns indicate that D-serine-associated microbial shifts co-varied with rumen metabolite changes; however, the correlations are statistical associations and should not be read as evidence of causal links.

## 4 Discussion

### 4.1 D-serine promotes the transition from liquid milk-based nutrition to solid feed intake in pre-weaning lambs

The most pronounced response to D-serine (D-Ser) supplementation in pre-weaning lambs was not an immediate change in body weight but rather an enhancement in the acceptance and consumption of starter feed. Lambs in the D-Ser group exhibited significantly greater average daily starter feed intake (ADFI) and cumulative starter feed intake, and the age at first voluntary starter consumption tended to be earlier than that in the control group. These findings suggest that D-Ser was associated primarily with the early phase of the dietary transition from liquid milk-based nutrition to solid feed. This transition is of critical importance for young ruminants: milk-derived nutrients largely bypass the rumen and enter the abomasum directly, whereas solid feed enters the rumen and provides the substrate basis for microbial colonization, fermentable substrate supply, and volatile fatty acid (VFA) production (Diao et al., 2019; Khan et al., 2016). Recent multi-omics and transcriptomic studies have further demonstrated that early introduction of solid feed not only reshapes ruminal microbial composition but also promotes ruminal tissue maturation through microbial metabolite-host epithelial transcriptional interactions (Bian et al., 2024; Malmuthuge et al., 2019; Sun et al., 2024; Sun et al., 2021). Therefore, the increase in starter feed intake should be viewed as an early nutritional transition response that may contribute to, but does not alone prove, ruminal functional development.

This finding also accounts for the discrepancy between growth performance and feed intake responses observed in the present study. Although final body weight (FBW), total weight gain (TWG), and average daily gain (ADG) were numerically higher in the D-Ser group, none of these differences reached statistical significance, suggesting that D-Ser cannot be defined as a direct growth-promoting additive at the current experimental stage. A more plausible interpretation is that starter feed intake was more sensitive to D-Ser supplementation, with the intake response preceding measurable changes in body weight. Before weaning, lamb growth is still largely determined by milk-derived nutrient supply and individual developmental variation, while the present sample size also limits the detection of growth responses. Thus, the current evidence supports improved starter intake rather than enhanced growth performance.

The novelty of the present study lies in evaluating D-Ser within the developmental window of ruminal microecology during the pre-weaning period. D-amino acids have long been considered to have limited nutritional utilization value in animals, and research on their biological roles has been markedly less extensive than that on L-form functional amino acids (Friedman, 1999; Genchi, 2017). In microbial systems, however, D-amino acids participate in cell wall remodeling and function as regulators of community interactions and environmental adaptation (Aliashkevich et al., 2018). The ruminal microbiota of pre-weaning lambs is still in an assembly phase characterized by high plasticity and heightened sensitivity to nutritional signals (Furman et al., 2020). Within this context, the promotion of starter feed intake by D-Ser suggests that its effects may not derive from direct energy provision alone but rather from changes at the ruminal microbial metabolic-host developmental interface. However, because the present design did not include an isonitrogenous L-serine, glycine, or total amino acid control, nor a graded D-serine dose series, the specificity of the response to D-serine versus a high amino acid load remains to be resolved.

Several lines of indirect evidence nonetheless argue that the observed responses are, at least in part, specific to D-serine rather than a generic consequence of a high amino acid load. In our earlier comparative study in suckling Hu lambs (Zhang et al., 2022), four functional amino acids were supplemented at comparable per-kilogram doses (2 g·kg^−1^ BW·d^−1^ for D-serine, glutamine, and citrulline; 0.5 g·kg^−1^ BW·d^−1^ for guanidinoacetic acid); a purely load-driven effect would be expected to produce broadly similar responses, yet the outcomes were compound-specific, with D-serine eliciting the largest increase in average daily gain (+35.30%), glutamine a smaller increase (+23.53%), and citrulline and guanidinoacetic acid no significant change, and with each amino acid generating a distinct plasma signature dominated by its own related metabolites. D-serine also possesses an enantiomer-specific mechanism that is not shared by a generic amino acid load, acting as an endogenous co-agonist at the glycine site of the NMDA receptor to promote growth hormone release, consistent with the elevated plasma GH and IGF-I previously observed with D-serine supplementation (Zhang et al., 2022). Moreover, the up-regulation of serine-derived metabolites such as L-N-carboxymethylserine in the present rumen metabolome is consistent with specific metabolic handling of the supplemented D-serine rather than its use as undifferentiated nitrogen. These observations are supportive rather than conclusive, however, and definitive attribution of the effects to D-serine will require isonitrogenous L-serine, glycine, and total-amino-acid controls, a graded D-serine dose series, and D- versus L-serine enantiomer-contrasting experiments.

### 4.2 D-serine induces mucosa-prioritized ruminal maturation rather than non-specific organ enlargement

The present study indicates that D-serine was associated with tissue-layer-selective morphological changes in the rumen. Without a significant difference in slaughter body weight between groups, the D-Ser group exhibited increases in rumen fresh weight, rumen weight-to-live weight ratio, and ruminal volume. More biologically meaningful, however, were the concurrent increases in papilla length, width, density, and epithelial thickness, whereas muscular layer thickness remained unchanged. This morphological pattern indicates that D-serine was associated with preferential expansion of the absorptive surface constituted by papillae and epithelium rather than generalized ruminal wall thickening. The ruminal papillae and epithelial layer are the primary sites for volatile fatty acid (VFA) absorption, transport, and local metabolism, but the present measurements demonstrate morphological development rather than complete functional maturation (Ghaffari et al., 2025; Pokhrel and Jiang, 2024; Steele et al., 2016).

This mucosa-prioritized morphological development is consistent with the fundamental principles governing early ruminal development. Ruminal epithelial and papillary development is driven primarily by fermentation end-products, particularly chemical signals such as butyrate and propionate, whereas muscular layer development depends more on physical stimuli including digesta fill, friction, and mechanical distension (Khan et al., 2016; Sander et al., 1959). In the present trial, lambs were still in the pre-weaning period with solid feed intake only recently established, so the intensity of physical stimulation in the rumen was limited. The absence of significant muscular thickening therefore supports a cautious interpretation: D-serine-associated fermentation and microbial-metabolite changes coincided with mucosal morphological development rather than proving complete ruminal functional maturation.

The elevated absolute concentration of butyrate provides a plausible explanation for this observation. Butyrate serves both as a major energy substrate for ruminal epithelial cells and as a signaling molecule that regulates epithelial cell proliferation, differentiation, and apoptosis (Malhi et al., 2013; Niwińska et al., 2017; Penner et al., 2011; Wu, 2009). Unlike direct supplementation with butyrate salts, the present study observed increased butyrate production and improved mucosal morphology through D-serine intervention, suggesting that D-serine may be associated with microbial metabolic conversion in the rumen and an endogenous chemical environment conducive to epithelial development. Recent studies have also demonstrated that early ruminal microorganisms and their metabolites can promote ruminal development through microbiota-metabolite-host epithelium interactions (Bian et al., 2024; Sun et al., 2024). The value of D-serine, therefore, may lie not in serving as a conventional nutritional substrate that directly supplies energy, but rather in its potential role as a D-amino acid signal associated with the early ruminal microecosystem and mucosal morphological development.

It should be noted that mucosal thickening does not necessarily equate to complete functional maturation. Excessive butyrate or premature high-concentrate stimulation may disrupt terminal differentiation of keratinocytes and induce abnormal keratinization of the ruminal epithelium (Zhang K. et al., 2024). Future studies should therefore examine the expression of MCT1, DRA, NHE, HMGCS2, IGF-1R, and keratinization differentiation markers to confirm whether the D-serine-associated mucosal growth is accompanied by stable absorptive, barrier, and metabolic functions. Overall, the key scientific contribution of the present study is the morphological evidence that D-serine was associated with mucosa-prioritized rumen development rather than non-specific organ hypertrophy; functional maturation remains to be verified by transporter, barrier, and epithelial metabolic indicators.

### 4.3 Propionate-enriched fermentation provides a metabolic foundation for improved ruminal function

A key finding of the present study is that D-serine did not merely elevate the concentration of a single fermentation end-product but rather altered VFA carbon-flow partitioning while enhancing overall fermentative capacity. Following D-serine supplementation, total VFA (TVFA) concentration rose to 77.42 mmol·L^−1^, with absolute concentrations of acetate, propionate, and butyrate all significantly increased; among these, the increase in propionate was the most pronounced. Concurrently, the molar proportion of propionate increased from 21.41 to 25.11%, and the acetate-to-propionate ratio (A: P) decreased from 2.90 to 2.44. VFA production depends on the coupled activities of ruminal microbial degradation of fermentable carbohydrates, glycolytic flux, and short-chain fatty acid biosynthetic pathways (Bergman, 1990; Hackmann, 2023; Hackmann and Firkins, 2015). The elevated TVFA observed in this study can therefore be interpreted as enhanced substrate utilization flux in ruminal fermentation, and the increased propionate proportion further indicates that the additional fermentative energy was not distributed evenly among all acid end-products but was preferentially channeled toward the glucogenic VFA.

Propionate enrichment represents the most metabolically significant change in this fermentation pattern. As young ruminants transition from milk-based nutrition to solid feed, glucose supply becomes increasingly dependent on hepatic gluconeogenesis, and rumen-derived propionate is the principal VFA precursor for gluconeogenesis, entering the tricarboxylic acid cycle via the propionyl-CoA–methylmalonyl-CoA–succinyl-CoA pathway to participate in glucose synthesis (Aschenbach et al., 2010; Larsen and Kristensen, 2013). Unlike acetate, which primarily serves peripheral oxidative energy supply and fatty acid synthesis, propionate provides a more direct link between ruminal fermentation and host glucose provision. The decrease in the A: P ratio signifies a shift in fermentation pattern from an acetate-dominant type toward a glucogenic type, representing an important metabolic signal of improved energy retention efficiency (Hackmann and Firkins, 2015; Reynolds et al., 2003). Propionate production also constitutes a significant reducing-equivalent sink within the rumen and may compete with methanogenesis for a portion of metabolic hydrogen (Jeong et al., 2024; Ungerfeld, 2020). However, because methane emissions were not directly measured in this study, this interpretation should be confined to mechanistic inference regarding fermentation hydrogen disposition rather than being stated as an emission-reduction conclusion.

Changes in butyrate provide an additional layer of explanation for ruminal mucosal development. D-serine increased the absolute concentration of butyrate from 7.42 to 8.96 mmol·L^−1^, yet the molar proportion of butyrate remained stable, indicating that the fermentation was not forced toward a single butyrate-dominant endpoint but rather that butyrate supply increased against the backdrop of enhanced overall fermentative flux. Butyrate is the preferentially utilized energy substrate by ruminal epithelial cells and serves as an important local signal driving papillary growth, epithelial cell proliferation, and the maturation of short-chain fatty acid absorptive capacity (Ghaffari et al., 2025; Malhi et al., 2013; Penner et al., 2011; Zhuang et al., 2024). These findings align with the increases in ruminal papilla length, width, density, and epithelial thickness observed in this study. D-serine-induced fermentation changes thus exhibit a clear dual orientation: increased propionate strengthens host glucogenic energy supply, while increased butyrate supports ruminal mucosal development.

The novelty of these results lies in the fact that D-serine was associated not with exogenous supplementation of a single end-product but with a rearrangement of the endogenous fermentation profile. Conventional butyrate salts primarily provide butyrate stimulation, with limited systemic modulation of TVFA and the A: P ratio (Górka et al., 2011; Niwińska et al., 2017). Ionophores can increase the propionate proportion, but typically rely on selective antimicrobial activity to reshape the fermentation ecology (Russell and Rychlik, 2001). In contrast, D-serine simultaneously elevated TVFA, increased propionate proportion, decreased the A: P ratio, and enhanced absolute butyrate supply, suggesting that its effects may arise from ruminal microbial recognition, utilization, or biotransformation of the D-amino acid to modulate carbon-flow partitioning. D-amino acids possess a dual identity as both substrates and signaling molecules in microbial ecology (Aliashkevich et al., 2018). The present study extends this understanding to the ruminal developmental stage of pre-weaning ruminants, suggesting that D-serine may act as a microbial metabolic modulator that provides a propionate-enriched metabolic foundation while concurrently supporting mucosal morphological development.

### 4.4 D-serine is associated with limited shifts in candidate functional taxa while preserving overall community stability

The central finding at the microbial community level was that D-serine did not induce a global drift in the ruminal microbiota but was associated with limited shifts in taxa linked to fermentation and mucosal interaction. Following D-serine supplementation, neither alpha diversity nor beta diversity based on Bray–Curtis dissimilarity differed significantly between groups, indicating that community richness, evenness, and overall structure remained stable. Concurrently, the relative abundance of *Bacteroidota* increased from 46.27 to 52.92%, and LEfSe analysis identified the enrichment of *Shuttleworthia*, *Erysipelotrichaceae*_UCG-006, *Erysipelotrichaceae*_UCG-009, *Corynebacterium*, and *Sutterella* in the D-serine group. These results indicate that D-serine did not broadly perturb the ruminal microecosystem but rather produced modest, directionally coherent shifts within the existing community framework. For pre-weaning lambs, this characteristic is of particular importance. The early ruminal microbiota is in a highly plastic assembly phase, during which aggressive interventions may disrupt colonization continuity; a more desirable nutritional strategy should promote functional development while avoiding reductions in community stability (Furman et al., 2020; Ghaffari et al., 2025; Li et al., 2023; Yáñez-Ruiz et al., 2015).

The increase in *Bacteroidota* provides a possible microbiological explanation for the propionate-enriched fermentation pattern described above. *Bacteroidota* and its *Prevotellaceae*-related taxa perform key functions in the rumen, including complex carbohydrate degradation, conversion of glycolytic intermediates, and substrate provision to the succinate pathway, thereby constituting a microbial basis linking polysaccharide utilization to propionate production (Betancur-Murillo et al., 2023; Hackmann, 2023; Stewart et al., 2019; Trautmann et al., 2023). The increase in *Bacteroidota* abundance in the present study was directionally consistent with the elevated propionate concentration, increased propionate molar proportion, and decreased acetate-to-propionate ratio, suggesting that D-serine may have shifted fermentation end-products toward glucogenic volatile fatty acids (VFA) through changes in the ecological weight of carbohydrate-utilizing and succinate-related metabolic taxa. It is noteworthy that dominant genera such as *Prevotella*, *Prevotella*_7, and *Rikenellaceae*_RC9_gut_group did not change significantly, indicating that the *Bacteroidota* expansion was not driven by overgrowth of a single dominant genus but more likely resulted from coordinated adjustments among multiple low-to-moderate abundance taxa within the phylum. This magnitude of change is more consistent with mild microbial shifts during early ruminal development than with broad microbiota remodeling.

The genus-level biomarkers enriched in the D-serine group further suggested candidate microbial directions of D-serine regulation. *Shuttleworthia* belongs to acid-producing taxa, and its enrichment is consistent with the elevated butyrate absolute concentration and improved ruminal papillae development observed in this study. Given that butyrate-producing potential is relatively common among various *Lachnospiraceae*-related gastrointestinal bacteria, this result suggests that D-serine may have enhanced local butyrate supply, although strain-level verification remains necessary (Downes et al., 2002; Vital et al., 2014). The enrichment of *Erysipelotrichaceae*_UCG-006 and _UCG-009 corresponded to the upregulation of isodeoxycholic acid and the enrichment of the secondary bile acid biosynthesis pathway in the metabolome. *Erysipelotrichaceae* has been associated with bile acid transformation and host metabolic signaling, suggesting that the effects of D-serine may extend beyond VFA production to include microbiota-associated bile acid metabolism (Collins et al., 2023; Kaakoush, 2015).

The changes in *Corynebacterium* and *Sutterella* provide candidate microbial clues for the amino acid metabolism and mucosal interaction effects of D-serine. Representative species of *Corynebacterium* possess strong amino acid synthesis, transformation, and export capabilities, and *Corynebacterium glutamicum* has been utilized in D-amino acid synthesis and export studies; therefore, its enrichment has a plausible connection to the enrichment of glycine, serine, and threonine metabolism and the upregulation of L-N-carboxymethylserine observed in this study (Aliashkevich et al., 2018; Stäbler et al., 2011). *Sutterella* is a mucosal-associated commensal bacterium characterized by epithelial adhesion and low-inflammatory interaction, and its enrichment may help explain the local microecological context underlying the mucosa-prioritized morphological development of the rumen following D-serine treatment (Bian et al., 2024; Hiippala et al., 2016; Sun et al., 2024). It should be noted, however, that 16S rRNA gene sequencing provides only genus-level evidence and cannot directly infer strain-level function or causality. Accordingly, these genera are more appropriately regarded as functional candidate markers rather than confirmed effector strains.

The enrichment of *Alloprevotella* and *Prevotellaceae*_NK3B31_group in the control group should not be simplistically interpreted as unfavorable taxa. Both belong to *Prevotellaceae*-related clades that typically participate in saccharide substrate utilization and short-chain fatty acid production. Following D-serine treatment, the relative decline of these taxa more likely reflects ecological niche redistribution among different functional microbial groups rather than disruption of community homeostasis. Combined with the stability of alpha and beta diversity and the PICRUSt2-predicted pathways being concentrated in fundamental metabolic processes such as sugar metabolism, nucleotide biosynthesis, and cofactor synthesis, these observations suggest changes in predicted functional potential rather than measured microbial activity. Because PICRUSt2 infers function from 16S phylogeny, its results can only serve as supporting evidence for metabolic directionality and require further validation through metagenomic, metatranscriptomic, and stable isotope tracing approaches (Douglas et al., 2020).

Overall, the microecological response to D-serine in the pre-weaning rumen appears modest but directionally coherent: rather than broadly suppressing microbial populations or directly supplementing a specific fermentation end-product to modify the ruminal environment, D-serine was associated with a higher relative weight of taxa linked to carbohydrate fermentation, butyrate supply, amino acid transformation, bile acid metabolism, and mucosal interaction while preserving community stability. This finding suggests that D-amino acids may participate in the regulation of ruminal microbial metabolism, thereby providing microbiological evidence for early nutritional intervention strategies targeting ruminal development in young ruminants.

### 4.5 Metabolomic profiles suggest possible serine-related processing of exogenous D-serine in the rumen

Untargeted metabolomics provided evidence of ruminal metabolomic responses associated with exogenous D-serine. Following D-serine treatment, the ruminal fluid metabolic profiles were clearly separated in the orthogonal partial least squares discriminant analysis (OPLS-DA), yielding 248 differential metabolites, of which 176 were upregulated in the D-serine group. These differential metabolites were concentrated in amino acid-related pathways, including glycine, serine, and threonine metabolism; alanine, aspartate, and glutamate metabolism; cysteine and methionine metabolism; and arginine biosynthesis. The Kyoto Encyclopedia of Genes and Genomes (KEGG) enrichment results suggest that D-serine was associated with altered nitrogenous-substrate metabolism and amino acid carbon-skeleton pathways within the ruminal lumen, although these untargeted metabolomic data do not directly trace the metabolic fate of the supplemented D-serine (Kanehisa et al., 2023).

The marked upregulation of L-N-carboxymethylserine constitutes a putative metabolomic clue for serine-related derivative formation in this study. This metabolite exhibited a log2FC of 4.576 in the D-serine group, indicating that serine-related derivative formation was substantially amplified. The conventional view has often regarded D-amino acids as enantiomers poorly utilized by ruminants (Friedman, 1999); however, microbial responses to D-amino acids are far from passive tolerance. D-Amino acids can participate in bacterial cell wall remodeling, biofilm regulation, and community adaptation, and may serve as signaling molecules or metabolic substrates within microbial ecosystems (Aliashkevich et al., 2018; Cava et al., 2011). The elevation of L-N-carboxymethylserine, together with the enrichment of glycine, serine, and threonine metabolism, is consistent with the possibility that exogenous D-serine entered or affected the serine-related metabolic pool of the ruminal microbiota, but it does not by itself prove direct microbial conversion.

The significance of these serine-related metabolic changes extends beyond the elevation of a single metabolite. The interconversion of serine and glycine serves as an important entry point for one-carbon metabolism, providing one-carbon units to the tetrahydrofolate cycle and thereby influencing nucleotide synthesis, the methionine cycle, and redox homeostasis (Ducker and Rabinowitz, 2017; Locasale, 2013). The concurrent enrichment of cysteine and methionine metabolism, arginine biosynthesis, and alanine, aspartate, and glutamate metabolism in the present study indicates that the effects of D-serine were associated with broader nitrogen flux redistribution and carbon skeleton rearrangement. Recent research has also demonstrated that gut microorganisms can efficiently consume and transform luminal amino acids, thereby influencing the host-available amino acid profile and nutritional homeostasis (Li et al., 2024). Considering the high-density anaerobic fermentation environment of the rumen, the present results support the interpretation that D-serine did not remain metabolically inert, but stable isotope tracing and chirality-targeted metabolomics are needed to verify its exact carbon and nitrogen fates.

The metabolomic results further suggest that the scope of D-serine-associated responses may extend beyond amino acid metabolism. Isodeoxycholic acid was significantly upregulated, accompanied by enrichment of the secondary bile acid biosynthesis pathway, suggesting that D-serine treatment altered the metabolic environment in a manner consistent with microbial metabolism. Bile acids can be transformed by gastrointestinal microorganisms through deconjugation, dehydroxylation, and epimerization reactions, and participate in energy metabolism, barrier function, and host immune regulation (Collins et al., 2023; Zhang B. et al., 2024). Accordingly, the metabolic effects of D-serine are more appropriately understood as a network response centered on serine-related metabolic changes, with concurrent engagement of amino acid, carbohydrate, and bile acid metabolism. This finding raises the possibility that D-amino acids in young ruminants may participate in the regulation of fermentation patterns and ruminal development through ruminal microecological processes.

It should be emphasized that both L-N-carboxymethylserine and isodeoxycholic acid currently represent putative identifications at Metabolomics Standards Initiative (MSI) Level 2 and have not been verified by standard co-elution and MS/MS co-fragmentation. According to metabolomics reporting standards, Level 2 evidence is sufficient to support biological inference but cannot substitute for structural confirmation and targeted quantification (Schymanski et al., 2014; Sumner et al., 2007). The bounded conclusion that can be drawn from this study is therefore as follows: exogenous D-serine supplementation was associated with serine-related metabolic shifts in the rumen, centered on serine derivative formation and amino acid metabolic pathway enrichment. The precise carbon and nitrogen fates, chiral conversion processes, and contributions of key microbial taxa remain to be elucidated through 13C/15N-labeled D-serine tracing, chirality-targeted metabolomics, and cultivation of key bacterial strains.

### 4.6 Tri-axial metabolism of amino acids, carbohydrates, and bile acids links microbiota remodeling to host rumen development

Metabolomic results of the present study indicated that the effects of D-serine on the rumen of suckling lambs were not confined to serine metabolism per se but were associated with three metabolic categories: amino acid, carbohydrate, and bile acid metabolism. The concurrent enrichment of glycine, serine, and threonine metabolism; cysteine and methionine metabolism; starch and sucrose metabolism; carbohydrate digestion and absorption; and secondary bile acid biosynthesis suggested that D-serine entering the rumen may have been associated with a broader spectrum of metabolic signals rather than passing through the rumen passively as an inert enantiomer (Aliashkevich et al., 2018; Kanehisa et al., 2023).

The amino acid metabolic axis represents the starting point of this process. The marked upregulation of L-N-carboxymethylserine provided putative metabolomic evidence consistent with possible D-serine-related processing within the rumen. Serine participates in nucleotide synthesis, the methionine cycle, and cellular redox homeostasis through glycine and one-carbon metabolism; therefore, the enhancement of this pathway likely reflects elevated anabolic activity of the ruminal microbiota (Ducker and Rabinowitz, 2017). The enrichment of *Corynebacterium* in the D-Ser group further supports this interpretation, as members of this genus possess robust amino acid synthesis, interconversion, and export capabilities and may contribute to the microbial metabolism of D-serine and its derivatives (Stäbler et al., 2011; Wendisch, 2020). Accordingly, D-serine is better characterized as a candidate functional amino acid substrate associated with ruminal microbial metabolism than as a poorly utilized D-amino acid in the conventional sense.

The carbohydrate metabolic axis may help explain the link between microbial compositional shifts and improved fermentation efficiency. The increased relative abundance of *Bacteroidota* in the D-Ser group, accompanied by the enrichment of starch and sucrose metabolism and carbohydrate digestion and absorption pathways, was consistent with enhanced polysaccharide degradation and fermentable substrate utilization capacity in the rumen. *Bacteroidota* and *Prevotella*-related taxa harbor extensive polysaccharide utilization loci and can contribute to propionate production via the succinate pathway (Betancur-Murillo et al., 2023; Stewart et al., 2019). This is consistent with the elevated total volatile fatty acid (TVFA) concentration, propionate absolute concentration, and propionate molar proportion observed in the present study. In other words, D-serine may have been associated with the coupling of microbial nitrogen metabolism and carbohydrate fermentation, directing a greater proportion of substrate flux toward propionate production and thereby improving the energetic efficiency of ruminal fermentation (Hackmann, 2023).

The bile acid metabolic axis constitutes one of the more novel findings of this study. The D-Ser group exhibited enrichment of the secondary bile acid biosynthesis pathway, significant upregulation of isodeoxycholic acid, and concurrent enrichment of *Erysipelotrichaceae*_UCG-006 and *Erysipelotrichaceae*_UCG-009. Previous studies have demonstrated that gastrointestinal microorganisms can remodel the bile acid pool through deconjugation, oxidoreduction, epimerization, and 7α-dehydroxylation reactions, and *Erysipelotrichaceae* has been linked to bile acid metabolism (Collins et al., 2023; Funabashi et al., 2020; Labbé et al., 2014). Although the present study cannot confirm that these genera directly produce isodeoxycholic acid, the concordance among microbial enrichment, pathway enrichment, and key metabolite upregulation suggests that bile acid metabolism may participate in the ruminal microecological response induced by D-serine. Secondary bile acids function not only as metabolites associated with lipid digestion but also as important signaling molecules that regulate energy metabolism, barrier function, and immune homeostasis through receptors such as FXR and TGR5 (Collins et al., 2023; Fleishman and Kumar, 2024). This finding extends the scope of D-serine action from intraluminal fermentation to microbiota-mediated host metabolic signaling.

Taken together, D-serine may first interact with functionally active ruminal microbiota and become associated with serine-related amino acid metabolism and microbial anabolism. Subsequently, enhanced microbial metabolic potential may be coupled with carbohydrate fermentation, increasing TVFA and propionate production. Concurrently, alterations in secondary bile acid metabolism may further contribute to mucosal barrier maintenance, energy metabolism, and host adaptive regulation. Recent studies in lambs have also shown that early-life ruminal microbe-derived metabolites can participate in the regulation of ruminal epithelial and muscular layer development (Bian et al., 2024; Sun et al., 2024). The scientific contribution of the present study lies in linking D-amino acid intervention, candidate microbiota-metabolite shifts, enhanced amino acid and carbohydrate metabolism, altered bile acid signaling, and preferential ruminal mucosal morphological development into a coherent hypothesis-generating framework. It should be noted, however, that the key metabolites identified in this study remain at Metabolomics Standards Initiative (MSI) Level 2 putative identification, and the causal relationships between the bile acid axis and the associated genera require further validation through reference standard confirmation, metagenomic or metatranscriptomic analyses, and stable isotope-labeled D-serine tracing experiments.

### 4.7 Microbiota–metabolite covariation network supports an integrative mode of action for D-serine

Correlation analysis between microbial taxa and metabolites further indicated that the ruminal effects of D-serine did not manifest as isolated changes in individual genera or single metabolites but rather as a coordinated response involving candidate functional taxa and key metabolites. Spearman correlation analysis was performed after centered log-ratio (CLR) transformation to mitigate compositional bias inherent in relative abundance data. The results showed that the genera enriched in the D-Ser group, *Shuttleworthia*, *Erysipelotrichaceae*_UCG-006, *Erysipelotrichaceae*_UCG-009, *Corynebacterium*, and *Sutterella*, were positively correlated with nine upregulated key metabolites overall, indicating that microbial shifts and metabolic profile changes following D-serine supplementation were directionally consistent. Correlation networks cannot establish causality; however, they facilitate identification of core genera and metabolic nodes that may participate in functional regulation (Faust and Raes, 2012; Layeghifard et al., 2017).

*Corynebacterium* was significantly correlated with multiple upregulated metabolites, suggesting its potential involvement in D-serine-associated ruminal amino acid metabolism. Representative species of this genus possess strong amino acid synthesis, interconversion, and export capabilities and can participate in the metabolism of serine and D-amino acids (Peters-Wendisch et al., 2005; Stäbler et al., 2011). Considered together with the marked upregulation of L-N-carboxymethylserine and the enrichment of glycine, serine, and threonine metabolism, the network position of *Corynebacterium* is consistent with the possibility that exogenous D-serine entering the rumen may be microbially processed and linked to amino acid metabolism and one-carbon metabolism. This further supports the characterization of D-serine as a candidate functional amino acid substrate for the ruminal microecosystem rather than a metabolically inert enantiomer (Aliashkevich et al., 2018).

The correlation results for *Sutterella* suggest that D-serine-associated metabolic changes may extend to the mucosal interface. *Sutterella* exhibits epithelial adhesion properties and can establish relatively stable commensal relationships with the intestinal mucosa (Hiippala et al., 2016). In the present study, this genus was positively correlated with multiple upregulated metabolites, echoing the morphological findings of preferential ruminal mucosal development. Recent lamb studies have also demonstrated that early-life ruminal microbe-derived metabolites can participate in the regulation of ruminal epithelial and muscular layer development (Bian et al., 2024; Sun et al., 2024). Therefore, *Sutterella* may represent a candidate genus linking intraluminal metabolic changes to host mucosal responses.

The strong correlations of *Erysipelotrichaceae*_UCG-009 with steroid and plant-derived terpenoid metabolites position the bile acid axis as an informative lead in this section. *Erysipelotrichaceae* has been associated with bile acid exposure, bile salt hydrolysis, and bile acid pool remodeling (Kaakoush, 2015; Labbé et al., 2014). Gastrointestinal microorganisms can generate secondary bile acids through deconjugation, oxidoreduction, epimerization, and 7α-dehydroxylation reactions (Collins et al., 2023; Funabashi et al., 2020; Kim et al., 2022). The concordance of isodeoxycholic acid upregulation, secondary bile acid biosynthesis pathway enrichment, and *Erysipelotrichaceae*-related taxa enrichment in this study suggests that D-serine may influence microbial bile acid metabolism in the rumen and downstream digestive tract. Bile acids not only participate in lipid digestion but also regulate energy metabolism, barrier function, and immune homeostasis through receptors such as FXR and TGR5 (Fleishman and Kumar, 2024). This finding raises the possibility that rumen development may involve bile acid-associated microbial-metabolite signaling beyond the traditional VFA-centered paradigm.

The enrichment of *Shuttleworthia* connects the covariation network to VFA changes. This genus belongs to the family *Lachnospiraceae*, members of which are known for saccharolytic fermentation and butyrate production (Meehan and Beiko, 2014; Vital et al., 2014). The elevated absolute concentration of butyrate observed in this study may provide an energy substrate and developmental signal for the ruminal epithelium, whereas the concurrent increases in propionate absolute concentration and molar proportion furnish a metabolic basis for host gluconeogenesis and improved energetic efficiency. Recent work has highlighted that ruminal short-chain fatty acid production is not determined by any single genus but is collectively shaped by substrate utilization, reducing equivalent allocation, and microbial interactions (Hackmann, 2023). This is consistent with the network results of the present study, indicating that D-serine modulates fermentation patterns primarily through a functional microbial consortium rather than through changes in the abundance of any individual genus.

Collectively, the possible mode of action of D-serine can be summarized as follows. Upon D-serine supplementation, ruminal genera associated with amino acid interconversion, acidogenic fermentation, bile acid metabolism, and mucosal interaction were selectively enriched. These genera formed covariation relationships with L-N-carboxymethylserine, isodeoxycholic acid, and multiple other upregulated metabolites, accompanying concurrent shifts in amino acid, carbohydrate, and bile acid metabolism. The resultant enhancement of TVFA and propionate production may provide metabolic support for ruminal mucosal development and the transition to solid feed intake. The scientific significance of these results lies in repositioning D-amino acids from conventionally regarded low-utilization substrates to candidate functional nutritional factors that may modulate the ruminal microecosystem and metabolic development of young ruminants.

It should be acknowledged that the covariation network constructed in the present study reveals only statistical associations among variables and is insufficient to establish direct causal relationships. Spearman correlation cannot distinguish among direct microbial biotransformation, indirect inter-microbial interactions, and common environmental drivers. Future studies should integrate metagenomic and metatranscriptomic analyses with stable isotope-labeled D-serine tracing to further track the metabolic flux of D-serine across the microbiota, metabolome, and host tissues. Key metabolite identifications should be upgraded to MSI Level 1 through reference standard confirmation, and isolation and cultivation of *Corynebacterium*, *Sutterella*, and *Erysipelotrichaceae*-related strains should be pursued to verify their functional contributions within this network (Surana and Kasper, 2017).

## 5 Conclusion

Dietary D-Ser supplementation at 2 g·kg^−1^ BW·d^−1^ improved starter intake and was associated with mucosa-prioritized morphological development of the rumen in pre-weaning Hu lambs. These effects were associated with higher TVFA production, a propionate-enriched fermentation profile, limited shifts in candidate functional bacterial taxa, and enrichment of amino acid-carbohydrate-bile acid metabolic signatures. The marked increase in L-N-carboxymethylserine is consistent with possible ruminal microbial processing of exogenous D-Ser, but direct transformation was not experimentally verified. D-Ser may therefore serve as a candidate functional amino acid for early-life rumen programming, although dose–response relationships, isonitrogenous amino acid controls, functional maturation markers, and causal microbial mechanisms require further validation.

## Data Availability

The raw microbial sequence data reported in this article have been deposited in the NCBI Sequence Read Archive (SRA) under BioProject accession PRJNA1467476 (https://www.ncbi.nlm.nih.gov/bioproject/PRJNA1467476). The original metabolomics dataset generated in this study has been deposited in OMIX, China National Centerunder under accession number OMIX017075 (https://ngdc.cncb.ac.cn/omix/release/OMIX017075).

## Footnotes

1
https://magic.novogene.com

2
https://cloud.metware.cn
